# Analysis of ocular adverse events associated with SNRIs

**DOI:** 10.3389/fphar.2026.1837592

**Published:** 2026-05-29

**Authors:** Zhengtai Sun, Yuting Liu, LiJuan Que

**Affiliations:** Department of Ophthalmology, The First Affiliated Hospital of Soochow University, Suzhou, China

**Keywords:** FDA adverse event reporting system, ocular adverse events, pharmacovigilance, serotonin-norepinephrine reuptake inhibitors, sex differences

## Abstract

**Background:**

Serotonin-norepinephrine reuptake inhibitors (SNRIs) are widely used in the treatment of neuropsychiatric disorders and chronic pain; however, their comprehensive ocular safety profile has not been systematically evaluated. This study aimed to investigate and compare the safety signals of ocular adverse events (AEs) associated with five major SNRIs (venlafaxine, duloxetine, desvenlafaxine, milnacipran, and levomilnacipran).

**Methods:**

This was a retrospective pharmacovigilance analysis. Ocular AEs associated with the SNRIs were extracted from the FAERS database (Q1 2004-Q4 2024). Disproportionality analyses were conducted using four algorithms, proportional reporting ratio (PRR), reporting odds ratio (ROR), Bayesian confidence propagation neural network (BCPNN), and multi-item gamma Poisson shrinker (MGPS), to identify statistically significant signals. Time-to-onset analysis and sex subgroup analyses were also performed.

**Results:**

A total of 5,860 SNRI-related ocular AEs were included. At the System Organ Class level, no significant positive signals of ocular AEs were observed. At the Preferred Term (PT) level, distinct risk profiles emerged for different SNRIs. Mydriasis was one of the strongest and most common signals for duloxetine, desvenlafaxine, and venlafaxine. Venlafaxine was also strongly associated with anisocoria and accommodation disorder. Vision blurred and visual impairment were the most frequently reported Preferred Term across all drugs. TTO analysis revealed that AEs occurred later for levomilnacipran (median 161 days) and venlafaxine (median 147 days), but earlier for desvenlafaxine (31 days) and milnacipran (11.5 days). Sex subgroup analyses revealed significant differences.

**Conclusion:**

Based on real-world data, this study identified drug-specific and sex-specific differences in the ocular safety signals of SNRIs. Mydriasis represents a common strong risk signal across multiple SNRIs.

## 1 Background

Serotonin and norepinephrine reuptake inhibitors (SNRIs) are first-line pharmacotherapies for various conditions, including major depressive disorder, generalized anxiety disorder, and chronic pain syndromes (Ohyama et al., 2025). Representative agents in this class include venlafaxine, duloxetine, desvenlafaxine, milnacipran, and its active enantiomer levomilnacipran (Lin et al., 2025). With their expanding clinical use, the safety profile of SNRIs has garnered continuous attention. While well-documented adverse effects predominantly involve the central nervous, cardiovascular, and gastrointestinal systems (Xiao. et al., 2023; Lin et al., 2025), potential rare or delayed adverse reactions necessitate ongoing monitoring and assessment in real-world settings.

Among various organ systems, the eye, as a delicate and highly specialized sensory organ, is particularly vulnerable to drug-induced adverse reactions, which may lead to irreversible visual impairment and significantly impact patients’ quality of life and treatment adherence (Santaella and Fraunfelder, 2007). Conventional clinical trials are often limited by sample size, follow-up duration, and population homogeneity, rendering them insufficient for comprehensively capturing such events. In recent years, pharmacovigilance studies utilizing large-scale spontaneous reporting databases have emerged as pivotal tools to address this gap. The U.S. Food and Drug Administration Adverse Event Reporting System (FAERS), the world’s largest database of drug safety reports, has become an essential resource for mining potential safety signals and conducting post-marketing safety reevaluation (Tan et al., 2024). Although previous studies leveraging FAERS have explored the overall adverse event profiles of SNRIs and preliminarily suggested potential associations between specific SNRIs (e.g., duloxetine, venlafaxine) and an increased risk of glaucoma (Le et al., 2024), existing research has largely focused on individual agents or specific ocular conditions. A systematic and comprehensive analysis of ocular adverse event signals across the entire SNRI class, along with comparative assessments, remains lacking. Therefore, this study aims to conduct a comprehensive analysis of the FAERS database to systematically assess, characterize, and compare the ocular safety signals of five SNRIs (venlafaxine, duloxetine, desvenlafaxine, milnacipran, and levomilnacipran), with the goal of enhancing clinical awareness and guiding future research.

## 2 Methods

### 2.1 Data source and collection

We conducted a retrospective pharmacovigilance assessment using the FAERS database, reviewing ocular AEs reports associated with SNRIs from Q1 2004 to Q4 2024. The FAERS database aggregates information from various sources, including demographic and administrative details (DEMO), adverse reactions (REAC), patient outcomes (OUTC), drug-specific information (DRUG), therapy timelines (THER), reporter entity details (RPSR), and indications for use (INDI). This information was utilized to classify AEs based on individual patient drug exposure. First, this study performed signal mining of all adverse events associated with serotonin-norepinephrine reuptake inhibitors (SNRIs) using the FAERS database, and extracted all preferred terms (PTs) along with their corresponding Medical Dictionary for Regulatory Activities (MedDRA) System Organ Classes (SOCs). Subsequently, based on the hierarchical structure of MedDRA (version 26.0), all PTs belonging to the SOC “Eye disorders” (SOC code: 10015919) were identified and defined as ocular adverse events, which were then subjected to subsequent statistical analyses, visualization, and safety assessment.

#### 2.1.1 Inclusion criteria

1. Use of an SNRI agent (venlafaxine, desvenlafaxine, milnacipran, levomilnacipran, or duloxetine).
2. Patient age ≥ 18 years.
3. Data recorded in the FAERS database.
4. AEs temporally associated with SNRI use, defined as occurrence during treatment or within a clinically plausible timeframe following drug administration. Temporal association was assessed based on the THER and the REAC, requiring that the treatment start date precede the adverse reaction onset date.

#### 2.1.2 Exclusion criteria

1. Duplicate reports: For multiple reports of the same adverse event in the same patient, only the initial report was retained. According to FDA recommendations, the PRIMARYID, CASEID, and FDA_DT fields are selected from the DEMO table to eliminate duplicate reports. The dataset is sorted by CASEID, FDA_DT, and PRIMARYID. For individual case safety reports (ICSRs) with the same CASEID, the report with the highest FDA_DT is retained. If multiple reports share the same CASEID and FDA_DT, the one with the highest PRIMARYID is selected.
2. Substantially missing data: Reports lacking at least two key elements, such as patient demographics (e.g., age, sex), drug information (e.g., dose), or adverse event details, were excluded.
3. Non-target drug association: Reports where the adverse event was clearly attributed to another drug, an underlying disease, or an external factor (e.g., surgical complication, trauma) without a direct link to SNRI use.
4. Ambiguous information: Reports with unclear or insufficiently detailed drug names or adverse event descriptions that precluded definitive assessment for study eligibility.

The detailed inclusion and exclusion process is shown in Supplementary Figure S1.

### 2.2 Signal detection data mining

Proportional Reporting Ratio (PRR), Reporting Odds Ratio (ROR), Bayesian Confidence Propagation Neural Network (BCPNN), and Multi-item Gamma Poisson Shrinker (MGPS) algorithms were applied to determine the statistical association between SNRI and ocular AEs. ROR, PRR, BCPNN, and MGPS are commonly used algorithms for disproportionality analysis, currently widely employed by the Medicines and Healthcare products Regulatory Agency (MHRA), the Netherlands Pharmacovigilance Center, the World Health Organization (WHO), and the FDA. ROR and PRR are frequentist (non-Bayesian) algorithms, with the advantage of correcting for bias in events with low reporting rates; PRR benefits from being less susceptible to underreporting of adverse events. Non-Bayesian (frequency-based) methods offer simple computation and high sensitivity but carry a high risk of false positives when adverse event numbers are low (Li et al., 2024). BCPNN and MGPS are Bayesian algorithms. BCPNN and MGPS are Bayesian disproportionality methods that tend to be more stable in sparse-data settings (Yang et al., 2025). Bayesian methods offer greater stability, account for uncertainty in small event sizes, reduce false alarm rates, and support higher-dimensional pattern recognition. However, they involve complex computations and exhibit relatively delayed signal detection. Therefore, this study employs a combined approach using multiple algorithms to leverage their respective strengths, broaden detection coverage, and validate results from multiple perspectives. This comprehensive methodology aims to identify more reliable safety signals (Tables 1, 2). In this study, four disproportionality analysis methods were used for signal detection, with the positive signal criteria defined as follows. For the ROR method, a positive signal was defined as a case count ≥3 and a lower limit of the 95% confidence interval (CI) for ROR > 1. For the PRR method, a positive signal was defined as a case count ≥3, PRR ≥ 2, and a chi-square statistic (χ^2^) ≥ 4. For the BCPNN method, a positive signal was defined as a lower limit of the 95% CI for the information component (IC-2SD) > 0. For the MGPS method, a positive signal was defined as a lower limit of the 95% CI for the empirical Bayesian geometric mean (EB05) ≥ 2. To enhance the robustness of signal detection, only drug–event combinations that simultaneously met the positive criteria for all four methods were considered final positive signals.

**TABLE 1:** Fourfold table of disproportionality analyses.

Medicine	Target adverse events reported	Other adverse events reported	Summation
Target drugs	a	b	a+b
Other drugs	c	d	c + d
Summation	a + c	b + d	N = a + b + c + d

**TABLE 2:** Four algorithms used for signal detection.

Algorithm	Equation	Criteria
ROR	ROR = ad/bc	Lower limit of 95% CI > 1, N ≥ 3
	95%CI = e^ln(ROR)±1.96(1/a+1/b+1/c+1/d)^0.5^^	
PRR	PRR = [a (c + d)]/[c (a + b)]	PRR ≥2, *χ* ^*2*^ ≥ 4, N ≥ 3
	*χ* ^*2*^ = [(ad-bc)^2^](a + b + c + d)/[(a+b) (c + d) (a+c) (b + d)]	
BCPNN	IC = log_2_a (a + b + c + d)/[(a + c) (a + b)]	IC025 > 0
	95% *CI* = E (IC) ± 2 [V(IC)]^0.5^	
MGPS	EBGM = a (a+b + c + d)/[(a + c) (a + b)]	EBGM05 > 2
	95% *CI* = e^ln(EBGM)±1.96(1/a+1/b+1/c+1/d)^0.5^^	

ROR: reporting odds ratio; PRR: proportional reported ratio; BCPNN: bayesian confidence propagation neural network; MGPS: multiple Gamma Poisson Shrinker; EBGM: empirical Bayesian geometric mean; CI: confidence interval; a: the number of reports containing both target drug and target adverse drug reaction; b: the number of reports containing the target adverse drug reaction with other medications (except the target drug); c: the number of reports containing the target drug with other adverse drug reactions (except the target events); d: the number of all reports.

### 2.3 Data calculation

Ocular AEs where SNRIs were the primary suspect (PS) drug were extracted. Values for the four-fold tables were calculated using respective formulas. The count data were described using case numbers and proportions.

### 2.4 Statistical analysis

Data cleaning and management were performed using R software, with visualizations generated using the ggplot2 package. Descriptive analyses summarized the clinical characteristics of patients experiencing ocular AEs associated with SNRIs. Time-to-onset (TTO) analysis and heatmap visualization were conducted to investigate the temporal patterns of ocular AE occurrence following SNRIs initiation. Subgroup analyses stratified by sex were performed to explore potential sex-based differences in SNRI-related ocular AEs.

## 3 Results

### 3.1 Baseline characteristics of AE reports

**FIGURE 1:**
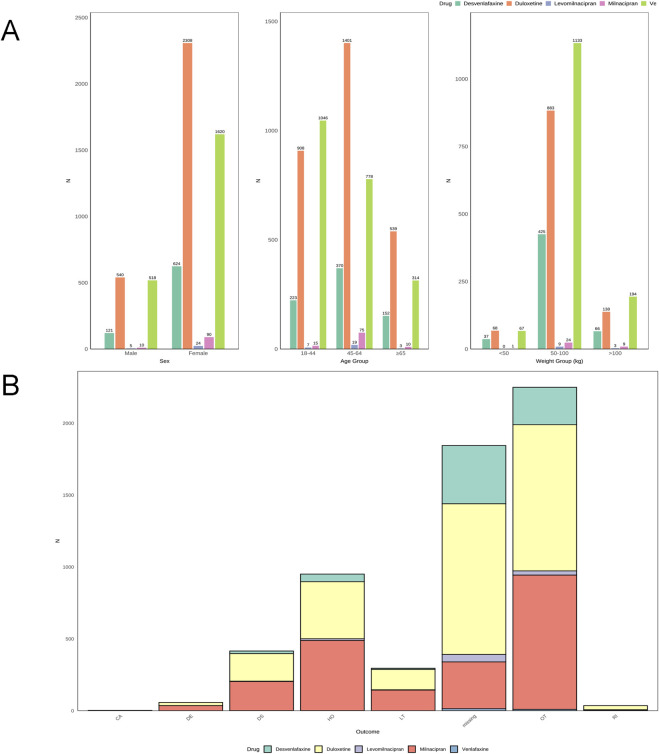
Demographic characteristics of ocular adverse events associated with serotonin-norepinephrine reuptake inhibitors **(A)** sex, age and weight; **(B)** outcome. For desvenlafaxine, duloxetine, levomilnacipran, and milnacipran, the predominant age group was 45–64 years, whereas venlafaxine-associated reports were most frequent among individuals aged 18–44 years. Females constituted the majority of reporters for all SNRIs; no male cases were reported for milnacipran, and venlafaxine had the relatively lowest female proportion (78.65%). Body weight across all SNRI groups was primarily concentrated in the 50–100 kg range, with venlafaxine showing the highest proportion (85.19%) and milnacipran the lowest (70.59%). Outcomes were predominantly classified as “other” or missing.

A total of 5,860 reports of ocular AEs associated with SNRIs were identified, comprising 745 for desvenlafaxine, 2,848 for duloxetine, 29 for levomilnacipran, 100 for milnacipran, and 2,138 for venlafaxine (Table 3). The predominant age group for desvenlafaxine, duloxetine, levomilnacipran, and milnacipran was 45–64 years, whereas venlafaxine-associated reports were most frequent among individuals aged 18–44 years (Figure 1A). Females constituted the majority of reporters for all SNRIs; no male cases were reported for milnacipran, and venlafaxine had the relatively lowest female predominance (78.65%) (Figure 1A). Overall, body weight across all SNRI groups was primarily concentrated in the 50–100 kg range, with venlafaxine showing the highest proportion in this category (85.19%) and milnacipran the lowest (70.59%) (Figure 1A). Regarding reporter occupation, the desvenlafaxine, duloxetine, and levomilnacipran groups were predominantly composed of consumers, physicians, and missing data. The United States was the primary reporting country among reports with available reporter country data across all five groups (93.9%, 68.13%, 83.33%, 100.00%, and 47.22%, respectively). Outcomes were predominantly classified as “other” or missing (Figure 1B). Fatal outcomes were reported only in the duloxetine and venlafaxine groups.

**TABLE 3:** Baseline characteristics.

Variable	Level	Desvenlafaxine	Duloxetine	Levomilnacipran	Milnacipran	Venlafaxine
n	​	745	2,848	29	100	2,138
Age (year)	​	51.84 ± 14.52	49.12 ± 14.32	51.92 ± 15.00	53.03 ± 10.11	44.91 ± 16.02
Age group (%)	≥65	97 (18.48)	137 (12.77)	1 (8.33)	5 (14.71)	165 (12.41)
​	18–44	150 (28.57)	394 (36.72)	4 (33.33)	7 (20.59)	701 (52.71)
​	45–64	278 (52.95)	542 (50.51)	7 (58.33)	22 (64.71)	464 (34.89)
Weight (kg)	​	75.24 ± 20.98	78.08 ± 21.80	83.44 ± 18.49	85.28 ± 29.37	0 (0.00)
Weight group (%)	<50	35 (6.67)	53 (4.94)	0 (0.00)	1 (2.94)	54 (4.06)
​	>100	65 (12.38)	137 (12.77)	3 (25.00)	9 (26.47)	143 (10.75)
​	50–100	425 (80.95)	883 (82.29)	9 (75.00)	24 (70.59)	1,133 (85.19)
Sex (%)	Female	446 (84.95)	881 (82.11)	11 (91.67)	34 (100.00)	1,046 (78.65)
​	Male	79 (15.05)	192 (17.89)	1 (8.33)	0 (0.00)	284 (21.35)
Occupation (%)	CN	287 (54.67)	601 (56.01)	5 (41.67)	18 (52.94)	744 (55.94)
​	HP	4 (0.76)	14 (1.30)	0 (0.00)	0 (0.00)	58 (4.36)
​	LW	0 (0.00)	4 (0.37)	0 (0.00)	0 (0.00)	57 (4.29)
​	MD	81 (15.43)	144 (13.42)	2 (16.67)	1 (2.94)	157 (11.80)
​	Missing	76 (14.48)	214 (19.94)	5 (41.67)	12 (35.29)	114 (8.57)
​	OT	67 (12.76)	54 (5.03)	0 (0.00)	1 (2.94)	152 (11.43)
​	PH	10 (1.90)	38 (3.54)	0 (0.00)	1 (2.94)	48 (3.61)
​	RN	0 (0.00)	4 (0.37)	0 (0.00)	1 (2.94)	0 (0.00)
Report country (%)	Argentina	0 (0.00)	1 (0.09)	0 (0.00)	0 (0.00)	0 (0.00)
​	Australia	1 (0.19)	6 (0.56)	0 (0.00)	0 (0.00)	4 (0.30)
​	Austria	0 (0.00)	4 (0.37)	0 (0.00)	0 (0.00)	7 (0.53)
​	Belgium	0 (0.00)	9 (0.84)	0 (0.00)	0 (0.00)	10 (0.75)
​	Brazil	19 (3.62)	16 (1.49)	0 (0.00)	0 (0.00)	10 (0.75)
​	Canada	9 (1.71)	60 (5.59)	0 (0.00)	0 (0.00)	67 (5.04)
​	China	0 (0.00)	1 (0.09)	0 (0.00)	0 (0.00)	3 (0.23)
​	Colombia	1 (0.19)	0 (0.00)	0 (0.00)	0 (0.00)	0 (0.00)
​	Not specified	0 (0.00)	3 (0.28)	2 (16.67)	0 (0.00)	2 (0.15)
​	Denmark	0 (0.00)	2 (0.19)	0 (0.00)	0 (0.00)	1 (0.08)
​	Egypt	0 (0.00)	1 (0.09)	0 (0.00)	0 (0.00)	0 (0.00)
​	Entity 1*	0 (0.00)	52 (4.85)	0 (0.00)	0 (0.00)	58 (4.36)
​	Estonia	0 (0.00)	0 (0.00)	0 (0.00)	0 (0.00)	1 (0.08)
​	Finland	0 (0.00)	0 (0.00)	0 (0.00)	0 (0.00)	4 (0.3)
​	France	0 (0.00)	33 (3.08)	0 (0.00)	0 (0.00)	110 (8.27)
​	Germany	0 (0.00)	18 (1.68)	0 (0.00)	0 (0.00)	122 (9.17)
​	Greece	0 (0.00)	2 (0.19)	0 (0.00)	0 (0.00)	3 (0.23)
​	Italy	0 (0.00)	4 (0.37)	0 (0.00)	0 (0.00)	8 (0.60)
​	Japan	0 (0.00)	15 (1.40)	0 (0.00)	0 (0.00)	5 (0.38)
​	Netherlands	0 (0.00)	4 (0.37)	0 (0.00)	0 (0.00)	33 (2.48)
​	New Zealand	0 (0.00)	0 (0.00)	0 (0.00)	0 (0.00)	2 (0.15)
​	Oman	0 (0.00)	1 (0.09)	0 (0.00)	0 (0.00)	0 (0.00)
​	Panama	1 (0.19)	0 (0.00)	0 (0.00)	0 (0.00)	0 (0.00)
​	Portugal	0 (0.00)	0 (0.00)	0 (0.00)	0 (0.00)	1 (0.08)
​	Puerto Rico	0 (0.00)	0 (0.00)	0 (0.00)	0 (0.00)	2 (0.15)
​	Slovenia	0 (0.00)	0 (0.00)	0 (0.00)	0 (0.00)	1 (0.08)
​	Spain	1 (0.19)	3 (0.28)	0 (0.00)	0 (0.00)	4 (0.30)
​	Sweden	0 (0.00)	2 (0.19)	0 (0.00)	0 (0.00)	29 (2.18)
​	Switzerland	0 (0.00)	4 (0.37)	0 (0.00)	0 (0.00)	4 (0.30)
​	Turkey	0 (0.00)	1 (0.09)	0 (0.00)	0 (0.00)	0 (0.00)
​	Ukraine	0 (0.00)	1 (0.09)	0 (0.00)	0 (0.00)	0 (0.00)
​	United Kingdom	0 (0.00)	99 (9.23)	0 (0.00)	0 (0.00)	211 (15.86)
​	United States	493 (93.9)	731 (68.13)	10 (83.33)	34 (100.00)	628 (47.22)
Outcome (%)	DE	0 (0.00)	9 (0.84)	0 (0.00)	0 (0.00)	15 (1.13)
​	DS	10 (1.90)	148 (13.79)	1 (8.33)	3 (8.82)	163 (12.26)
​	HO	35 (6.67)	144 (13.42)	1 (8.33)	6 (17.65)	247 (18.57)
​	LT	5 (0.95)	100 (9.32)	0 (0.00)	0 (0.00)	86 (6.47)
​	Missing	305 (58.10)	212 (19.76)	4 (33.33)	8 (23.53)	222 (16.69)
​	OT	170 (32.38)	431 (40.17)	6 (50.00)	15 (44.12)	594 (44.66)
​	RI	0 (0.00)	29 (2.70)	0 (0.00)	2 (5.88)	3 (0.23)
Death (%)	No	745 (100.00)	2,839 (99.16)	29 (100.00)	100 (100.00)	2,123 (98.87)
​	Yes	0 (0.00)	9 (0.84)	0 (0.00)	0 (0.00)	15 (1.13)

CN: consumer, HP: health professional, LW: lawyer, RN: registered nurse, MD: medical doctor, PH: pharmacist, OT: other, DE: death, DS: disability, HO: hospitalization, LT: Life-threatening, RI: required intervention to prevent permanent impairment/damage.

### 3.2 Primary system organ class-level signals

At the SOC level, no significant positive signals were detected for any of the five SNRIs (desvenlafaxine: 745 reports; duloxetine: 2,848 reports; levomilnacipran: 29 reports; milnacipran: 100 reports; venlafaxine: 2,138 reports) for ocular AEs (Table 4).

**TABLE 4:** Signal of adverse reactions at the SOC level in the eye for SNRIs.

Drug	a	ROR (95% Cl)	PRR (95% Cl)	PRR (χ^2^)	EBGM (95% Cl)	IC (95% Cl)
Desvenlafaxine	745	1.12 (1.04–1.20)	1.12 (1.04–1.20)	1.12 (9.20)	1.12 (1.05–1.19)	0.16 (-1.51 – 1.82)
Duloxetine	2,848	1.10 (1.06–1.14)	1.10 (1.06–1.14)	1.10 (25.81)	1.10 (1.06–1.13)	0.14 (-1.53 – 1.80)
Levomilnacipran	29	1.46 (1.01–2.12)	1.45 (1.00–2.10)	1.45 (4.14)	1.45 (1.07–1.98)	0.54 (-1.13 – 2.20)
Milnacipran	100	0.92 (0.75–1.12)	0.92 (0.75–1.12)	0.92 (0.72)	0.92 (0.78–1.09)	−0.12 (-1.79 – 1.55)
Venlafaxine	2,138	0.98 (0.94–1.02)	0.98 (0.94–1.02)	0.98 (0.90)	0.98 (0.95–1.02)	−0.03 (−1.70 – 1.64)

### 3.3 Primary risk signals at the PT level

At the PT level, the most frequently reported ocular AEs varied across SNRIs: for desvenlafaxine, duloxetine, and venlafaxine, the top three were vision blurred, visual impairment, and mydriasis (Tables 5-7); and for levomilnacipran, they were visual impairment, vision blurred, and keratitis (Table 8); for milnacipran, they were vision blurred, visual impairment and eye swelling (Table 9). Similar patterns were observed when ranked by signal strength: the strongest positive signals for desvenlafaxine were mydriasis, abnormal sensation in eye, and eye movement disorder; for duloxetine, they were mydriasis, photopsia, and eye movement disorder; for venlafaxine, they were anisocoria, accommodation disorder, and mydriasis; for levomilnacipran, visual impairment was the only significant positive signal; while no significant positive signals were identified for milnacipran.

**TABLE 5:** Signal of adverse reactions at the ocular PT level for desvenlafaxine.

PT	a	ROR (95%Cl)	PRR (95%Cl)	PRR (χ^2^)	EBGM (95% Cl)	IC (95% Cl)
Vision blurred	186	2.35 (2.03–2.71)	2.34 (2.03–2.71)	2.34 (143.10)	2.34 (2.07–2.64)	1.23 (-0.44–2.89)
Visual impairment	112	1.77 (1.47–2.13)	1.77 (1.47–2.13)	1.77 (37.39)	1.77 (1.51–2.06)	0.82 (-0.84–2.49)
Mydriasis	71	9.79 (7.75–12.37)	9.77 (7.73–12.35)	9.77 (553.63)	9.68 (7.96–11.78)	3.28 (1.61–4.94)
Eye disorder	31	1.87 (1.32–2.67)	1.87 (1.32–2.66)	1.87 (12.60)	1.87 (1.39–2.51)	0.90 (-0.76–2.57)
Photophobia	29	2.70 (1.87–3.88)	2.69 (1.87–3.88)	2.69 (30.83)	2.69 (1.98–3.65)	1.43 (-0.24–3.09)
Eye pain	25	0.90 (0.61–1.34)	0.90 (0.61–1.34)	0.90 (0.26)	0.90 (0.65–1.25)	−0.15 (-1.81 - 1.52)
Blindness	22	1.16 (0.76–1.76)	1.16 (0.76–1.76)	1.16 (0.49)	1.16 (0.82–1.65)	0.22 (-1.45–1.88)
Dry eye	22	0.94 (0.62–1.43)	0.94 (0.62–1.43)	0.94 (0.08)	0.94 (0.66–1.34)	−0.09 (-1.75 - 1.58)
Abnormal sensation in eye	18	7.48 (4.70–11.89)	7.48 (4.70–11.89)	7.48 (100.18)	7.42 (5.04–10.95)	2.89 (1.23–4.56)
Glaucoma	18	1.85 (1.16–2.94)	1.85 (1.16–2.94)	1.85 (7.00)	1.85 (1.25–2.72)	0.88 (-0.78–2.55)
Cataract	18	0.59 (0.37–0.93)	0.59 (0.37–0.93)	0.59 (5.17)	0.59 (0.40–0.87)	−0.76 (-2.43 - 0.90)
Eye movement disorder	16	4.35 (2.66–7.11)	4.35 (2.66–7.11)	4.35 (41.11)	4.34 (2.87–6.54)	2.12 (0.45–3.78)
Visual acuity reduced	13	0.64 (0.37–1.11)	0.64 (0.37–1.11)	0.64 (2.57)	0.64 (0.41–1.01)	−0.64 (-2.30 - 1.03)
Eye swelling	13	0.63 (0.36–1.08)	0.63 (0.37–1.08)	0.63 (2.85)	0.63 (0.40–0.99)	−0.67 (-2.33 - 1.00)
Diplopia	12	0.76 (0.43–1.33)	0.76 (0.43–1.33)	0.76 (0.95)	0.76 (0.47–1.21)	−0.40 (-2.07 - 1.26)
Ocular hyperaemia	9	0.40 (0.21–0.76)	0.40 (0.21–0.76)	0.40 (8.27)	0.40 (0.23–0.69)	−1.33 (-3.00 - 0.33)
Photopsia	9	2.23 (1.16–4.30)	2.23 (1.16–4.30)	2.23 (6.12)	2.23 (1.29–3.86)	1.16 (-0.51–2.82)
Eye irritation	9	0.40 (0.21–0.76)	0.40 (0.21–0.76)	0.40 (8.33)	0.40 (0.23–0.68)	−1.34 (-3.00 - 0.33)
Asthenopia	8	2.93 (1.46–5.87)	2.93 (1.46–5.87)	2.93 (10.14)	2.92 (1.64–5.23)	1.55 (-0.12–3.22)
Angle closure glaucoma	6	3.34 (1.50–7.44)	3.34 (1.50–7.44)	3.34 (9.78)	3.33 (1.7–6.51)	1.73 (0.07–3.40)
Ocular discomfort	6	1.22 (0.55–2.71)	1.22 (0.55–2.71)	1.22 (0.23)	1.22 (0.62–2.38)	0.28 (-1.38–1.95)
Lacrimation increased	6	0.37 (0.17–0.83)	0.37 (0.17–0.83)	0.37 (6.26)	0.38 (0.19–0.73)	−1.41 (-3.08 - 0.25)
Eye pruritus	6	0.37 (0.16–0.82)	0.37 (0.16–0.82)	0.37 (6.55)	0.37 (0.19–0.72)	−1.44 (-3.11 - 0.22)
Blepharospasm	5	1.54 (0.64–3.70)	1.54 (0.64–3.70)	1.54 (0.94)	1.54 (0.74–3.20)	0.62 (-1.05–2.29)
Eye haemorrhage	4	0.55 (0.2–1.45)	0.55 (0.20–1.45)	0.55 (1.51)	0.55 (0.24–1.24)	−0.87 (-2.54 - 0.79)
Visual field defect	4	0.85 (0.32–2.26)	0.85 (0.32–2.26)	0.85 (0.11)	0.85 (0.37–1.92)	−0.24 (-1.91 - 1.43)
Blindness transient	4	1.04 (0.39–2.78)	1.04 (0.39–2.78)	1.04 (0.01)	1.04 (0.46–2.37)	0.06 (-1.61–1.73)
Periorbital oedema	3	0.90 (0.29–2.80)	0.90 (0.29–2.80)	0.90 (0.03)	0.90 (0.35–2.32)	−0.15 (-1.82 - 1.52)
Metamorphopsia	3	2.75 (0.89–8.56)	2.75 (0.89–8.56)	2.75 (3.34)	2.75 (1.07–7.10)	1.46 (-0.21–3.13)
Myopia	3	2.44 (0.78–7.57)	2.44 (0.78–7.57)	2.44 (2.54)	2.43 (0.94–6.28)	1.28 (-0.39–2.95)

**TABLE 6:** Signal of adverse reactions at the ocular PT level for duloxetine.

PT	a	ROR (95% Cl)	PRR (95% Cl)	PRR (χ^2^)	EBGM (95% Cl)	IC (95% Cl)
Vision blurred	682	2.23 (2.06–2.4)	2.22 (2.06–2.39)	2.22 (453.79)	2.21 (2.07–2.35)	1.14 (-0.52–2.81)
Visual impairment	298	1.21 (1.08–1.36)	1.21 (1.08–1.36)	1.21 (10.96)	1.21 (1.1–1.33)	0.28 (-1.39–1.94)
Mydriasis	174	6.25 (5.38–7.26)	6.24 (5.37–7.26)	6.24 (746.91)	6.11 (5.39–6.93)	2.61 (0.95–4.28)
Eye pain	129	1.20 (1.01–1.43)	1.20 (1.01–1.43)	1.20 (4.28)	1.20 (1.04–1.39)	0.26 (-1.40–1.93)
Photophobia	120	2.89 (2.41–3.46)	2.89 (2.41–3.46)	2.89 (146.42)	2.87 (2.47–3.33)	1.52 (-0.15–3.19)
Diplopia	97	1.58 (1.29–1.93)	1.58 (1.29–1.92)	1.58 (20.32)	1.57 (1.33–1.86)	0.65 (-1.01–2.32)
Visual acuity reduced	91	1.16 (0.94–1.43)	1.16 (0.94–1.43)	1.16 (2.01)	1.16 (0.98–1.38)	0.21 (-1.45–1.88)
Dry eye	75	0.83 (0.66–1.04)	0.83 (0.66–1.04)	0.83 (2.74)	0.83 (0.68–1.00)	−0.27 (-1.94 - 1.39)
Eye disorder	71	1.10 (0.87–1.39)	1.10 (0.87–1.39)	1.10 (0.69)	1.10 (0.91–1.34)	0.14 (-1.52–1.81)
Eye swelling	65	0.81 (0.63–1.03)	0.81 (0.63–1.03)	0.81 (2.91)	0.81 (0.66–0.99)	−0.3 (-1.97 - 1.36)
Photopsia	63	4.07 (3.18–5.23)	4.07 (3.17–5.22)	4.07 (143.61)	4.02 (3.26–4.95)	2.01 (0.34–3.67)
Eye movement disorder	50	3.53 (2.67–4.66)	3.52 (2.67–4.66)	3.52 (89.14)	3.49 (2.76–4.41)	1.8 (0.14–3.47)
Glaucoma	50	1.32 (1.00–1.75)	1.32 (1.00–1.75)	1.32 (3.91)	1.32 (1.05–1.67)	0.4 (-1.26–2.07)
Blindness	47	0.64 (0.48–0.85)	0.64 (0.48–0.85)	0.64 (9.66)	0.64 (0.5–0.81)	−0.65 (-2.31 - 1.02)
Eye irritation	47	0.53 (0.4–0.71)	0.53 (0.4–0.71)	0.53 (19.48)	0.53 (0.42–0.68)	−0.91 (-2.58 - 0.75)
Ocular hyperaemia	44	0.50 (0.37–0.67)	0.50 (0.37–0.67)	0.50 (22.17)	0.50 (0.39–0.64)	−1.00 (-2.67 - 0.66)
Cataract	39	0.33 (0.24–0.45)	0.33 (0.24–0.45)	0.33 (53.79)	0.33 (0.25–0.43)	−1.61 (-3.27 - 0.06)
Retinal detachment	39	1.96 (1.43–2.69)	1.96 (1.43–2.69)	1.96 (18.3)	1.96 (1.5–2.55)	0.97 (-0.7–2.63)
Lacrimation increased	31	0.50 (0.35–0.71)	0.50 (0.35–0.71)	0.50 (15.67)	0.50 (0.37–0.67)	−1.00 (-2.67 - 0.66)
Visual field defect	30	1.64 (1.15–2.35)	1.64 (1.15–2.35)	1.64 (7.44)	1.64 (1.21–2.21)	0.71 (-0.96–2.38)
Miosis	30	1.49 (1.04–2.14)	1.49 (1.04–2.14)	1.49 (4.83)	1.49 (1.1–2.01)	0.57 (-1.09–2.24)
Blepharospasm	26	2.07 (1.4–3.04)	2.07 (1.4–3.04)	2.07 (14.17)	2.06 (1.49–2.84)	1.04 (-0.63–2.71)
Abnormal sensation in eye	26	2.78 (1.89–4.09)	2.78 (1.89–4.09)	2.78 (29.33)	2.76 (2–3.82)	1.47 (-0.20–3.13)
Vitreous floaters	24	1.15 (0.77–1.71)	1.15 (0.77–1.71)	1.15 (0.45)	1.15 (0.82–1.6)	0.20 (-1.47–1.86)
Ocular discomfort	24	1.25 (0.84–1.87)	1.25 (0.84–1.87)	1.25 (1.23)	1.25 (0.90–1.75)	0.33 (-1.34–1.99)
Angle closure glaucoma	23	3.32 (2.20–5.00)	3.32 (2.20–5.00)	3.32 (36.70)	3.28 (2.33–4.63)	1.72 (0.05–3.38)
Eye pruritus	22	0.35 (0.23–0.53)	0.35 (0.23–0.53)	0.35 (27.20)	0.35 (0.24–0.49)	−1.53 (-3.19 - 0.14)
Blindness transient	18	1.21 (0.76–1.92)	1.21 (0.76–1.92)	1.21 (0.65)	1.21 (0.82–1.78)	0.27 (-1.39–1.94)
Retinal tear	17	3.24 (2.01–5.23)	3.24 (2.01–5.23)	3.24 (25.96)	3.21 (2.15–4.79)	1.68 (0.01–3.35)
Asthenopia	17	1.60 (1.00–2.58)	1.60 (1.00–2.58)	1.60 (3.84)	1.60 (1.07–2.38)	0.68 (-0.99–2.35)

**TABLE 7:** Signal of adverse reactions at the ocular PT level for venlafaxine.

PT	a	ROR (95% Cl)	PRR (95% Cl)	PRR (χ^2^)	EBGM (95% Cl)	IC (95% Cl)
Vision blurred	424	1.64 (1.49–1.80)	1.63 (1.49–1.80)	1.63 (104.16)	1.63 (1.51–1.77)	0.71 (-0.96–2.37)
Mydriasis	279	12.12 (10.75–13.66)	12.09 (10.72–13.63)	12.09 (2,723.87)	11.64 (10.53–12.87)	3.54 (1.87–5.21)
Visual impairment	217	1.05 (0.92–1.20)	1.05 (0.92–1.20)	1.05 (0.46)	1.05 (0.94–1.17)	0.07 (-1.60–1.73)
Photophobia	116	3.32 (2.76–3.99)	3.32 (2.76–3.98)	3.32 (185.77)	3.29 (2.82–3.84)	1.72 (0.05–3.38)
Eye pain	104	1.15 (0.95–1.39)	1.15 (0.95–1.39)	1.15 (2.01)	1.15 (0.98–1.35)	0.20 (-1.47–1.87)
Diplopia	71	1.37 (1.08–1.73)	1.37 (1.08–1.73)	1.37 (7.05)	1.37 (1.13–1.66)	0.45 (-1.21–2.12)
Miosis	59	3.51 (2.71–4.54)	3.51 (2.71–4.54)	3.51 (104.54)	3.48 (2.81–4.31)	1.80 (0.13–3.46)
Blindness	45	0.73 (0.54–0.97)	0.73 (0.54–0.97)	0.73 (4.65)	0.73 (0.57–0.93)	−0.46 (-2.13 - .21)
Eye disorder	43	0.79 (0.59–1.07)	0.79 (0.59–1.07)	0.79 (2.31)	0.79 (0.62–1.02)	−0.33 (-2.00 - 1.33)
Eye movement disorder	41	3.43 (2.52–4.66)	3.43 (2.52–4.66)	3.43 (69.67)	3.40 (2.63–4.40)	1.77 (0.10–3.43)
Dry eye	35	0.46 (0.33–0.64)	0.46 (0.33–0.64)	0.46 (22.50)	0.46 (0.35–0.6)	−1.13 (-2.79 - 0.54)
Photopsia	35	2.67 (1.91–3.72)	2.67 (1.91–3.72)	2.67 (36.22)	2.65 (2.01–3.51)	1.41 (-0.26–3.07)
Visual acuity reduced	32	0.48(0.34–0.68)	0.48 (0.34–0.68)	0.48 (17.62)	0.48 (0.36–0.65)	−1.05 (-2.71–0.62)
Eye swelling	31	0.46 (0.32–0.65)	0.46 (0.32–0.65)	0.46 (19.86)	0.46 (0.34–0.62)	−1.12 (-2.79–0.54)
Glaucoma	31	0.97 (0.68–1.38)	0.97 (0.68–1.38)	0.97 (0.02)	0.97 (0.72–1.31)	−0.04 (-1.71–1.63)
Cataract	31	0.31 (0.22–0.44)	0.31 (0.22–0.44)	0.31 (47.73)	0.31 (0.23–0.42)	−1.69 (-3.35–0.02)
Accommodation disorder	24	16.96 (11.24–25.60)	16.96 (11.24–25.6)	16.96 (340.43)	16.07 (11.39–22.68)	4.01 (2.33–5.68)
Asthenopia	23	2.59 (1.72–3.90)	2.59 (1.72–3.90)	2.59 (22.18)	2.57 (1.82–3.63)	1.36 (-0.30–3.03)
Eyelid oedema	21	0.82 (0.54–1.26)	0.82 (0.54–1.26)	0.82 (0.80)	0.82 (0.58–1.18)	−0.28 (-1.95 - 1.39)
Angle closure glaucoma	19	3.25 (2.07–5.11)	3.25 (2.07–5.11)	3.25 (29.25)	3.22 (2.21–4.71)	1.69 (0.02–3.36)
Retinal tear	19	4.31 (2.74–6.78)	4.31 (2.74–6.78)	4.31 (47.59)	4.26 (2.92–6.23)	2.09 (0.42–3.76)
Blepharospasm	19	1.79 (1.14–2.81)	1.79 (1.14–2.81)	1.79 (6.59)	1.79 (1.22–2.60)	0.84 (-0.83–2.50)
Ocular hyperaemia	18	0.24 (0.15–0.38)	0.24 (0.15–0.38)	0.24 (42.69)	0.24 (0.16–0.36)	−2.04 (-3.71 - −0.38)
Visual field defect	16	1.04 (0.63–1.69)	1.04 (0.63–1.69)	1.04 (0.02)	1.04 (0.69–1.56)	0.05 (-1.61–1.72)
Ocular hypertension	16	5.03 (3.07–8.25)	5.03 (3.07–8.25)	5.03 (50.79)	4.96 (3.28–7.50)	2.31 (0.64–3.98)
Abnormal sensation in eye	15	1.90 (1.14–3.15)	1.90 (1.14–3.15)	1.90 (6.34)	1.89 (1.24–2.89)	0.92 (-0.75–2.59)
Lacrimation increased	14	0.27 (0.16–0.45)	0.27 (0.16–0.45)	0.27 (28.15)	0.27 (0.17–0.42)	−1.90 (-3.57–−0.24)
Ocular discomfort	14	0.87 (0.51–1.47)	0.87 (0.51–1.47)	0.87 (0.28)	0.87 (0.56–1.35)	−0.20 (-1.87–1.46)
Pupil fixed	14	3.83 (2.26–6.50)	3.83 (2.26–6.50)	3.83 (28.93)	3.80 (2.44–5.90)	1.92 (0.26–3.59)
Anisocoria	11	19.95 (10.83–36.75)	19.95 (10.83–36.75)	19.95 (185.16)	18.72 (11.23–31.22)	4.23 (2.55–5.91)

**TABLE 8:** Signal of adverse reactions at the ocular PT level for levomilnacipran.

PT	a	ROR (95% Cl)	PRR (95% Cl)	PRR (χ^2^)	EBGM (95% Cl)	IC (95% Cl)
Visual impairment	10	5.31 (2.85–9.90)	5.27 (2.83–9.82)	5.27 (34.63)	5.27 (3.13–8.87)	2.40 (0.73–4.07)
Vision blurred	6	2.53 (1.13–5.64)	2.52 (1.13–5.62)	2.52 (5.51)	2.52 (1.29–4.93)	1.33 (-0.34–3.00)
Keratitis	1	18.68 (2.63–132.81)	18.66 (2.62–132.68)	18.66 (16.71)	18.65 (3.61–96.27)	4.22 (2.55–5.89)
Photophobia	1	3.10 (0.44–22.02)	3.10 (0.44–22.01)	3.10 (1.42)	3.10 (0.60–15.98)	1.63 (-0.04–3.30)
Eye swelling	1	1.62 (0.23–11.49)	1.62 (0.23–11.48)	1.62 (0.23)	1.62 (0.31–8.34)	0.69 (-0.98–2.36)
Eye irritation	1	1.47 (0.21–10.43)	1.47 (0.21–10.42)	1.47 (0.15)	1.47 (0.28–7.57)	0.55 (-1.12–2.22)
Retinal detachment	1	6.51 (0.92–46.27)	6.50 (0.92–46.23)	6.50 (4.66)	6.50 (1.26–33.56)	2.70 (1.03–4.37)
Mydriasis	1	4.56 (0.64–32.39)	4.55 (0.64–32.37)	4.55 (2.77)	4.55 (0.88–23.50)	2.19 (0.52–3.86)
Eye pain	1	1.21 (0.17–8.57)	1.21 (0.17–8.57)	1.21 (0.03)	1.21 (0.23–6.22)	0.27 (-1.40–1.94)
Cataract	1	1.09 (0.15–7.76)	1.09 (0.15–7.76)	1.09 (0.01)	1.09 (0.21–5.64)	0.13 (-1.54–1.80)
Ocular discomfort	1	6.78 (0.95–48.18)	6.77 (0.95–48.14)	6.77 (4.92)	6.77 (1.31–34.94)	2.76 (1.09–4.43)
Ocular hypertension	1	33.91 (4.77–241.23)	33.88 (4.76–241.00)	33.88 (31.87)	33.84 (6.55–174.76)	5.08 (3.41–6.75)
Dry eye	1	1.43 (0.20–10.16)	1.43 (0.20–10.16)	1.43 (0.13)	1.43 (0.28–7.37)	0.52 (-1.15–2.18)
Angle closure glaucoma	1	18.55 (2.61–131.87)	18.53 (2.61–131.75)	18.53 (16.57)	18.52 (3.59–95.59)	4.21 (2.54–5.88)
Eye haemorrhage	1	4.56 (0.64–32.42)	4.56 (0.64–32.40)	4.56 (2.78)	4.56 (0.88–23.52)	2.19 (0.52–3.86)

**TABLE 9:** Signal of adverse reactions at the ocular PT level for milnacipran.

PT	a	ROR (95% Cl)	PRR (95% Cl)	PRR (χ^2^)	EBGM (95% Cl)	IC (95% Cl)
Vision blurred	40	3.10 (2.27–4.24)	3.09 (2.26–4.22)	3.09 (56.59)	3.09 (2.38–4.01)	1.63 (-0.04–3.29)
Visual impairment	12	1.16 (0.66–2.05)	1.16 (0.66–2.05)	1.16 (0.27)	1.16 (0.72–1.87)	0.22 (-1.45–1.88)
Eye swelling	4	1.19 (0.45–3.17)	1.19 (0.45–3.17)	1.19 (0.12)	1.19 (0.52–2.70)	0.25 (-1.42–1.92)
Eye pain	3	0.66 (0.21–2.06)	0.66 (0.21–2.06)	0.66 (0.51)	0.66 (0.26–1.71)	−0.59 (-2.26 - 1.08)
Ocular hyperaemia	3	0.81 (0.26–2.52)	0.81 (0.26–2.52)	0.81 (0.13)	0.81 (0.31–2.09)	−0.30 (-1.97 - 1.37)
Mydriasis	3	2.51 (0.81–7.80)	2.51 (0.81–7.79)	2.51 (2.73)	2.51 (0.97–6.48)	1.33 (-0.34–3.00)
Dry eye	2	0.53 (0.13–2.10)	0.53 (0.13–2.10)	0.53 (0.86)	0.53 (0.16–1.68)	−0.93 (-2.59 - 0.74)
Visual acuity reduced	2	0.61 (0.15–2.43)	0.61 (0.15–2.43)	0.61 (0.51)	0.61 (0.19–1.94)	−0.72 (-2.39 - 0.95)
Photophobia	2	1.14 (0.28–4.55)	1.14 (0.28–4.55)	1.14 (0.03)	1.14 (0.36–3.63)	0.19 (-1.48–1.85)
Eye pruritus	2	0.75 (0.19–3.01)	0.75 (0.19–3.01)	0.75 (0.16)	0.75 (0.24–2.40)	−0.41 (-2.08 - 1.25)
Blepharospasm	2	3.77 (0.94–15.10)	3.77 (0.94–15.09)	3.77 (4.07)	3.77 (1.18–12.03)	1.91 (0.25–3.58)
Eye movement disorder	2	3.33 (0.83–13.32)	3.33 (0.83–13.31)	3.33 (3.25)	3.33 (1.04–10.61)	1.73 (0.07–3.40)
Eye haemorrhage	2	1.68 (0.42–6.70)	1.68 (0.42–6.70)	1.68 (0.55)	1.68 (0.53–5.35)	0.74 (-0.92–2.41)
Lacrimation increased	1	0.38 (0.05–2.72)	0.38 (0.05–2.72)	0.38 (0.99)	0.38 (0.07–1.98)	−1.38 (-3.05 - 0.28)
Visual acuity reduced transiently	1	33.17 (4.65–236.83)	33.16 (4.64–236.79)	33.16 (31.02)	32.98 (6.37–170.84)	5.04 (3.36–6.72)
Blindness unilateral	1	0.86 (0.12–6.10)	0.86 (0.12–6.10)	0.86 (0.02)	0.86 (0.17–4.43)	−0.22 (-1.89 - 1.45)
Visual field defect	1	1.30 (0.18–9.23)	1.30 (0.18–9.23)	1.30 (0.07)	1.30 (0.25–6.70)	0.38 (-1.29–2.05)
Glaucoma	1	0.63 (0.09–4.47)	0.63 (0.09–4.47)	0.63 (0.22)	0.63 (0.12–3.25)	−0.67 (-2.33 - 1.00)
Amaurosis	1	11.06 (1.55–78.65)	11.05 (1.55–78.64)	11.05 (9.13)	11.04 (2.14–56.98)	3.46 (1.79–5.14)
Myopia	1	4.98 (0.7–35.41)	4.98 (0.70–35.4)	4.98 (3.18)	4.98 (0.96–25.68)	2.32 (0.65–3.98)
Photopsia	1	1.52 (0.21–10.81)	1.52 (0.21–10.81)	1.52 (0.18)	1.52 (0.30–7.85)	0.61 (-1.06–2.27)
Eyelid margin crusting	1	4.72 (0.66–33.53)	4.72 (0.66–33.53)	4.72 (2.93)	4.72 (0.91–24.33)	2.24 (0.57–3.91)
Oscillopsia	1	37.45 (5.24–267.59)	37.44 (5.24–267.54)	37.44 (35.24)	37.21 (7.18–192.86)	5.22 (3.54–6.90)
Abnormal sensation in eye	1	2.53 (0.36–17.99)	2.53 (0.36–17.99)	2.53 (0.93)	2.53 (0.49–13.06)	1.34 (-0.33–3.01)
Vitreous floaters	1	1.14 (0.16–8.08)	1.14 (0.16–8.08)	1.14 (0.02)	1.14 (0.22–5.87)	0.19 (-1.48–1.85)
Diplopia	1	0.39 (0.05–2.74)	0.39 (0.05–2.74)	0.39 (0.97)	0.39 (0.07–1.99)	−1.37 (-3.04 - 0.29)
Eye irritation	1	0.27 (0.04–1.91)	0.27 (0.04–1.91)	0.27 (1.98)	0.27 (0.05–1.39)	−1.89 (-3.56 - −0.22)
Altered visual depth perception	1	12.51 (1.76–89.01)	12.51 (1.76–89)	12.51 (10.56)	12.48 (2.42–64.47)	3.64 (1.97–5.31)
Eye discharge	1	1.21 (0.17–8.56)	1.21 (0.17–8.56)	1.21 (0.04)	1.21 (0.23–6.22)	0.27 (-1.40–1.94)
Symblepharon	1	37.94 (5.31–271.11)	37.93 (5.31–271.06)	37.93 (35.72)	37.69 (7.27–195.38)	5.24 (3.55–6.92)

### 3.4 Time-to-onset analysis

**FIGURE 2:**
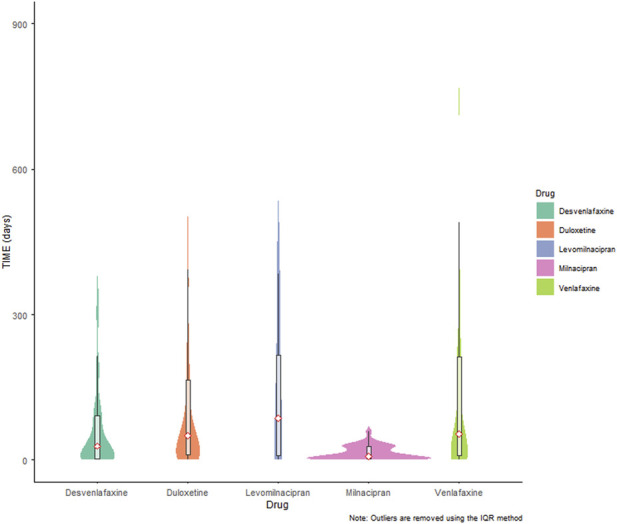
Violin plot of the onset time of ocular adverse events with SNRIs. The median onset times for desvenlafaxine, duloxetine, levomilnacipran, milnacipran, and venlafaxine were 31 days, 61 days, 161 days, 11.5 days, and 147 days, respectively.

**FIGURE 3:**
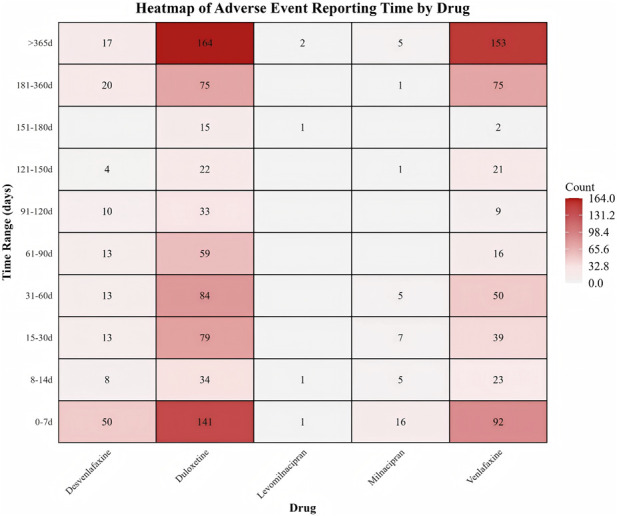
Heatmap of the onset time of ocular adverse events with SNRIs. Duloxetine and venlafaxine both showed higher frequencies of adverse event reports during the early treatment phase and long-term use. Among them, duloxetine had the highest number of reports (164 cases) beyond 365 days, with an overall reporting frequency significantly higher than that of other drugs. The overall levels of adverse event reports for levomilnacipran and milnacipran were relatively low.

TTO analysis revealed significant differences in the onset time of ocular AEs among the five SNRIs. Levomilnacipran (median: 161 days) and venlafaxine (median: 147 days) exhibited considerably longer median onset times compared to the other three agents. In contrast, desvenlafaxine (median: 31 days) and milnacipran (median: 11.5 days) were associated with earlier onset (Figure 2). Duloxetine demonstrated a broad distribution of onset times (Figure 3), with substantial numbers of AEs occurring both within the first week and after 1 year of treatment.

### 3.5 Subgroup analysis by sex

At the SOC level, no significant positive signals were detected for SNRIs in either the female or male subgroups (Tables 10, 11). However, at the PT level, sex-specific differences emerged. In the desvenlafaxine group, the strongest positive signals among females were mydriasis, abnormal sensation in eye, and halo vision (Supplementary Table S1), whereas among males, they were abnormal sensation in eye, mydriasis, and photopsia (Supplementary Table S2). For duloxetine, the top three positive signals in females were mydriasis, photopsia, and eye movement disorder (Supplementary Table S3), while in males, they were dysmetropsia, altered visual depth perception, and vitreous detachment (Supplementary Table S4). In the levomilnacipran group, only visual impairment and vision blurred showed significant signals in the female subgroup (Supplementary Table S5), with no significant signals observed in males (Supplementary Table S6). Similarly, in the milnacipran group, only vision blurred was significant in females (Supplementary Table S7), with no signals in males (Supplementary Table S8). For venlafaxine, the strongest signals in females were anisocoria, mydriasis, and ocular hypertension (Supplementary Table S9), whereas in males, they were vitreous degeneration, retinopathy hypertensive, and vitreous opacities (Supplementary Table S10).

**TABLE 10:** Signal of adverse reactions at the SOC level in the eye for SNRIs in female population.

Drug	a	ROR (95% Cl)	PRR (95% Cl)	PRR (χ^2^)	EBGM (95% Cl)	IC (95% Cl)
Desvenlafaxine	624	1.09 (1.01–1.18)	1.09 (1.01–1.18)	1.09 (4.96)	1.09 (1.02–1.17)	0.13 (-1.54–1.79)
Duloxetine	2,308	1.09 (1.05–1.14)	1.09 (1.04–1.13)	1.09 (16.68)	1.09 (1.05–1.13)	0.12 (-1.54–1.79)
Levomilnacipran	24	1.57 (1.04–2.36)	1.55 (1.03–2.33)	1.55 (4.78)	1.55 (1.1–2.18)	0.63 (-1.04–2.30)
Milnacipran	90	0.90 (0.73–1.1)	0.90 (0.73–1.11)	0.90 (1.09)	0.90 (0.75–1.07)	−0.16 (-1.82 - 1.51)
Venlafaxine	1,620	0.96 (0.92–1.01)	0.96 (0.92–1.01)	0.96 (2.30)	0.96 (0.92–1.00)	−0.05 (-1.72 - 1.61)

**TABLE 11:** Signal of adverse reactions at the SOC level in the eye for SNRIs in male population.

Drug	a	ROR (95% Cl)	PRR (95% Cl)	PRR (χ^2^)	EBGM (95% Cl)	IC (95% Cl)
Desvenlafaxine	121	1.04 (0.87–1.25)	1.04 (0.87–1.24)	1.04 (0.19)	1.04 (0.89–1.21)	0.06 (-1.61–1.72)
Duloxetine	540	1.01 (0.93–1.10)	1.01 (0.93–1.10)	1.01 (0.10)	1.01 (0.94–1.09)	0.02 (-1.65–1.68)
Levomilnacipran	5	1.03 (0.43–2.50)	1.03 (0.43–2.50)	1.03 (0.00)	1.03 (0.49–2.16)	0.04 (-1.63–1.72)
Milnacipran	10	0.77 (0.41–1.44)	0.77 (0.41–1.44)	0.77 (0.67)	0.77 (0.46–1.30)	−0.37 (-2.04 - 1.30)
Venlafaxine	518	0.97 (0.89–1.05)	0.97 (0.89–1.05)	0.97 (0.62)	0.97 (0.9–1.04)	−0.05 (-1.72 - 1.62)

## 4 Discussion

This study provides a comprehensive, comparative pharmacovigilance analysis of ocular AEs associated with five commonly used SNRIs, leveraging large-scale real-world data from the FAERS database. A total of 5,860 ocular AE reports were identified, and signal mining was conducted using multiple disproportionality methods. Key findings include: (1) While no significant signals were detected at the SOC level, whereas obvious positive signals presented at the PT level; (2) Heterogeneity exists in the ocular AE profiles among different SNRIs, with disturbances of vision blurred and visual impairment being the most prominent; (3) TTO of ocular AEs varied significantly across SNRIs; (4) Sex-stratified analyses suggested sex-specific differences in certain ocular AE signals. The findings are intended solely for hypothesis generation. These findings supplement the existing evidence base on the comprehensive ocular safety profile of SNRIs and provide valuable insights for clinical practice.

This study clearly suggested both similarities and differences in the ocular safety profiles of various SNRIs. Similarities are evident in that vision blurred and visual impairment were the most frequently reported PTs for all SNRIs, consistent with known common adverse reactions (Constable et al., 2022). Furthermore, desvenlafaxine, duloxetine, and venlafaxine each exhibited strong signals for mydriasis, with venlafaxine exhibited strong signals for accommodation disorder and anisocoria. These signals collectively suggest that SNRIs may interfere with ocular autonomic regulation. All mechanistic interpretations presented in this study are hypothesis-generating only. The noradrenergic system plays a crucial role in modulating the pupillary dilator muscle (causing mydriasis) and the ciliary muscle (involved in accommodation) (Sander, 2017; Jakobsen et al., 2023). By inhibiting norepinephrine reuptake, SNRIs may enhance sympathetic tone, leading to sustained mydriasis and accommodative dysfunction (Kiel et al., 2022; Behera et al., 2025). Clinically, mydriasis can result in photophobia and glare, while accommodative dysfunction directly causes blurred near vision, which may represent an important pathophysiological basis for patient-reported vision blurred. Therefore, for SNRI users complaining of visual issues, clinicians should proactively inquire about symptoms like photophobia or difficulty with near vision and consider simple examinations of pupillary light reflex and accommodative function. The strong signal for photopsia associated with duloxetine is noteworthy. Photopsia, typically perceived as flashes of light, may be related to retinal or vitreous traction (Sebag, 2018). This signal raises concern about potential retinal or vitreous effects of SNRIs, particularly duloxetine, with case reports suggesting possible visual disturbances (Martins et al., 2024). Differences were observed in that milnacipran and levomilnacipran yielded fewer and weaker positive signals, whereas signals for venlafaxine, duloxetine, and desvenlafaxine were more numerous and robust. This heterogeneity may stem from several factors. First, SNRIs differ in their ratios of serotonin to norepinephrine reuptake inhibition potency (Aldosary et al., 2022). Venlafaxine, for instance, exhibits more potent norepinephrine reuptake inhibition at higher doses, which may explain its stronger pupillary-related signals. Second, contributions from active metabolites vary; venlafaxine is metabolized to desvenlafaxine, itself an active SNRI (Sunil et al., 2020).

The TTO analysis further highlights inter-drug variability. The prolonged median onset times for levomilnacipran and venlafaxine suggested that their ocular AEs may have a delayed onset, extended clinical monitoring (Chokkakula et al., 2026). Conversely, the earlier onset for milnacipran and desvenlafaxine indicated a need for vigilance early in treatment. Duloxetine’s bimodal TTO distribution, with peaks both early (within 1 week) and late (after 1 year), suggests that risk monitoring should span the entire treatment duration. The early peak may be directly related to the initial pharmacological action of the drug (e.g., acute reactions triggered by serotonin and norepinephrine reuptake inhibition) (Xi et al., 2024); whereas the late peak may be associated with adaptive neuromodulatory changes after long-term use, metabolite accumulation, or progression of comorbid conditions. These temporal insights are crucial for developing individualized patient education and monitoring plans.

Subgroup analysis yielded important findings. The ocular risk signal profiles of SNRIs differed markedly between male and female patients. For instance, among females, mydriasis was the predominant signal for duloxetine and desvenlafaxine; among males, strong signals for duloxetine included dysmetropsia and altered visual depth perception. Venlafaxine-associated safety signals were entirely different between sexes: females predominantly exhibited pupillary abnormalities (anisocoria, mydriasis) and elevated intraocular pressure, while males showed signals related to vitreous degeneration and hypertensive retinopathy. These differences may be attributable to several factors. First, sex-based pharmacokinetic differences (Frackiewicz et al., 2000), such as variations in body weight, body fat percentage, and hepatic enzyme activity, could lead to differential drug exposure and plasma concentrations. Second, sex hormones (e.g., estrogen, testosterone) may modulate the sensitivity of central and ocular neurotransmitter systems, influencing responses to SNRIs (Liqiang and Kun, 2024). Additionally, sociobehavioral factors, such as potential differences in symptom reporting tendencies between men and women, may introduce bias in spontaneous reporting data. Notably, no significant signals were detected in the male subgroups for milnacipran and levomilnacipran, which is likely attributable to the very low utilization of these medications among males. These findings highlight the necessity of accounting for sex differences in drug safety monitoring and clinical practice, and future research should further investigate the underlying biological mechanisms.

Pharmacovigilance analyses based on the FAERS database should fully acknowledge inherent biases of spontaneous reporting systems. Underreporting is an inevitable major limitation, which precludes the calculation of true adverse event incidence and biases absolute risk estimation (Xia et al., 2025). Reporting behaviors are easily influenced by external stimuli such as drug safety alerts and media coverage, which may trigger a sudden surge in case reports and distort real risk trend assessment. Notoriety bias is also non-negligible (Reeves et al., 2025). Newly approved, popular, and publicly concerned drugs tend to have inflated reporting rates, undermining direct cross-drug comparison. Such reporting disparities may further induce channel bias in the comparison of SNRIs. Differences in marketing duration, clinical application range, indications, patient demographics, and clinicians’ risk awareness create unequal pathways for adverse events to be documented in FAERS. For example, venlafaxine and duloxetine yield far more reports than the other three SNRIs. This discrepancy largely reflects their longer marketing history, larger exposed population, and wider clinical use, rather than inherent differences in drug safety (Jiang X et al., 2023). Additionally, clinical attention to venlafaxine withdrawal syndrome and duloxetine-related hyponatremia may cause selective overreporting of relevant events. When interpreting inter-drug signal differences, report number and signal strength should be standardized by prescription volume or population exposure. Full consideration of non-pharmacologically driven channel bias is required to avoid misleading conclusions. The high proportion of female reports in this study introduces prominent gender-related reporting bias and complicates signal interpretation. Gender disparities in reporting may distort the estimation of signal magnitude for specific drugs and adverse events (Xu et al., 2025). The proportion of female reports was relatively high in the present study. Females have higher medication exposure or stronger reporting willingness, overall disproportionality analysis may overestimate adverse event risks in females while underestimating genuine signals in males. Such bias also interferes with cross-drug and cross-event comparisons (Zhu et al., 2025). Female-dominated reporting may be confounded by indications, concomitant medications, and sociobehavioral factors, such as higher tendency in females to seek medical advice and report symptoms. Detected signals may therefore reflect confounding factors rather than pure pharmacological effects, highlighting the necessity of gender-stratified analysis.

In spontaneous reporting-based pharmacovigilance, non-significant signals at the SOC level coupled with detectable signals at the PT level are common and logically reasonable, which stems from the hierarchical MedDRA structure and disproportionality analysis methodology (Shin et al., 2021). SOC represents broad organ system categories covering numerous subordinate PTs. When a drug only increases reports of a few specific PTs, its signal will be diluted by abundant irrelevant low-frequency events within the same SOC, making ROR and PRR fail to reach statistical thresholds. By contrast, PT-level analysis targets individual clinical manifestations and sensitively captures specific adverse reaction patterns. This hierarchical difference indicates localized, item-specific safety risks rather than extensive systemic organ impacts. In result interpretation, priority should be given to PT-level positive signals. Combined assessment based on biological plausibility and other evidence helps prevent the omission of potential ocular safety risks.

These findings carry several clinical implications. First, the distinct time-to-onset profiles suggest tiered monitoring: milnacipran and desvenlafaxine warrant early vigilance (within the first month), duloxetine requires both early and long-term follow-up (beyond 1 year) due to its bimodal distribution, and venlafaxine or levomilnacipran need prolonged observation given their delayed median onset. Second, for high-risk patients, such as those with narrow-angle glaucoma, diabetic retinopathy, pre-existing vitreoretinal diseases, or occupations demanding clear vision (e.g., drivers), duloxetine, venlafaxine, and desvenlafaxine should be used with caution, whereas milnacipran and levomilnacipran, which generated fewer ocular signals, may represent relatively safer alternatives. Third, the strong photopsia signal for duloxetine raises the possibility of vitreoretinal traction; thus, any duloxetine-treated patient complaining of flashing lights should undergo prompt ophthalmologic evaluation (including dilated fundoscopy) to rule out retinal tears. These practical strategies can inform prescribing decisions and ocular safety monitoring in routine clinical practice.

This study has inherent limitations. First, spontaneous reporting databases like FAERS were subject to underreporting, selective reporting, and incomplete information, which may affect the precision of risk signal quantification. Second, as a retrospective observational study, causality between SNRIs and ocular AEs cannot be established. Third, FAERS data do not permit calculation of incidence rates, confounding factors (e.g., comorbid conditions like diabetes or hypertension, concomitant medications) and exposure differences cannot be fully controlled; depression itself or common comorbidities might independently increase ocular disease risk. Fourth, the limited number of reports for certain drugs (e.g., levomilnacipran) may have resulted in insufficient statistical power to detect true but weak signals.

## 5 Conclusion

In summary, the signals of ocular AEs associated with SNRIs exhibited both drug-specific and sex-specific characteristics, with mydriasis emerging as a common strong signal across multiple agents. These findings complement the limitations of traditional clinical trials and provide clinicians with real-world evidence-based safety information. Given the increasing clinical utilization of SNRIs, strengthened monitoring and awareness of their ocular adverse effects are essential for protecting patients’ visual health and supporting treatment adherence.

## Data Availability

The original contributions presented in the study are included in the article/Supplementary Material, further inquiries can be directed to the corresponding author.

## Supplementary material

SUPPLEMENTARY FIGURE S1 Flowchart Note: FAERS data (2004Q1–2024Q4) were deduplicated, yielding 745 reports for desvenlafaxine, 2,848 for duloxetine, 29 for levomilnacipran, 100 for milnacipran, and 2,138 for venlafaxine. ROR, PRR, BCPNN, and MGPS were used for disproportionality analysis.
