# Supplementary material for: Analysis of ocular adverse events associated with SNRIs
Source: Front Pharmacol. 2026 May 29;17:1837592. doi: 10.3389/fphar.2026.1837592 (PMC13260375; doi:10.3389/fphar.2026.1837592)
Supplement: Supplementary file 1 [file Table1.docx]

Supplementary Table 1 Ocular adverse reactions female subjects treated with desvenlafaxine in PT level

| PT | a | ROR(95%Cl) | PRR(95%Cl) | PRR($\text{χ}^{\text{2}}$) | EBGM(95%Cl) | IC(95%Cl) |
| --- | --- | --- | --- | --- | --- | --- |
| Vision blurred | 152 | 2.21(1.89 - 2.60) | 2.21(1.88 - 2.59) | 2.21(100.36) | 2.20(1.93-2.52) | 1.14(-0.53 - 2.81) |
| Visual impairment | 94 | 1.68(1.37 - 2.06) | 1.68(1.37 - 2.06) | 1.68(25.88) | 1.68(1.42-1.99) | 0.75(-0.92 - 2.41) |
| Mydriasis | 61 | 9.80(7.61 - 12.62) | 9.78(7.59 - 12.59) | 9.78(474.66) | 9.67(7.82-11.94) | 3.27(1.61 - 4.94) |
| Eye disorder | 28 | 1.87(1.29 - 2.71) | 1.87(1.29 - 2.71) | 1.87(11.28) | 1.87(1.37-2.55) | 0.90(-0.77 - 2.57) |
| Photophobia | 21 | 2.21(1.44 - 3.39) | 2.21(1.44 - 3.39) | 2.21(13.82) | 2.20(1.54-3.15) | 1.14(-0.53 - 2.81) |
| Dry eye | 20 | 0.88(0.57 - 1.37) | 0.88(0.57 - 1.37) | 0.88(0.31) | 0.88(0.61-1.28) | -0.18(-1.84 - 1.49) |
| Eye pain | 19 | 0.74(0.47 - 1.16) | 0.74(0.47 - 1.16) | 0.74(1.77) | 0.74(0.51-1.08) | -0.44(-2.10 - 1.23) |
| Blindness | 18 | 1.19(0.75 - 1.89) | 1.19(0.75 - 1.89) | 1.19(0.56) | 1.19(0.81-1.76) | 0.25(-1.41 - 1.92) |
| Glaucoma | 16 | 1.88(1.15 - 3.06) | 1.88(1.15 - 3.06) | 1.88(6.52) | 1.87(1.24-2.82) | 0.91(-0.76 - 2.57) |
| Abnormal sensation in eye | 15 | 6.68(4.02 - 11.11) | 6.68(4.02 - 11.10) | 6.68(71.77) | 6.63(4.33-10.14) | 2.73(1.06 - 4.40) |
| Cataract | 15 | 0.55(0.33 - 0.91) | 0.55(0.33 - 0.91) | 0.55(5.67) | 0.55(0.36-0.83) | -0.87(-2.54 - 0.79) |
| Eye movement disorder | 14 | 4.78(2.83 - 8.08) | 4.78(2.82 - 8.08) | 4.78(41.55) | 4.75(3.06-7.38) | 2.25(0.58 - 3.92) |
| Eye swelling | 11 | 0.56(0.31 - 1.01) | 0.56(0.31 - 1.01) | 0.56(3.84) | 0.56(0.34-0.92) | -0.84(-2.51 - 0.83) |
| Eye irritation | 9 | 0.42(0.22 - 0.81) | 0.42(0.22 - 0.81) | 0.42(7.16) | 0.42(0.24-0.73) | -1.25(-2.91 - 0.42) |
| Diplopia | 9 | 0.72(0.37 - 1.38) | 0.72(0.37 - 1.38) | 0.72(1.02) | 0.72(0.41-1.24) | -0.48(-2.15 - 1.18) |
| Visual acuity reduced | 8 | 0.49(0.25 - 0.99) | 0.49(0.25 - 0.99) | 0.49(4.13) | 0.50(0.28-0.88) | -1.01(-2.68 - 0.65) |
| Ocular hyperaemia | 7 | 0.35(0.17 - 0.74) | 0.35(0.17 - 0.74) | 0.35(8.28) | 0.35(0.19-0.66) | -1.50(-3.17 - 0.17) |
| Asthenopia | 7 | 2.72(1.30 - 5.72) | 2.72(1.30 - 5.72) | 2.72(7.60) | 2.72(1.46-5.05) | 1.44(-0.23 - 3.11) |
| Angle closure glaucoma | 6 | 3.53(1.58 - 7.88) | 3.53(1.58 - 7.88) | 3.53(10.84) | 3.52(1.80-6.89) | 1.82(0.15 - 3.48) |
| Ocular discomfort | 6 | 1.35(0.61 - 3.01) | 1.35(0.61 - 3.01) | 1.35(0.54) | 1.35(0.69-2.64) | 0.43(-1.23 - 2.10) |
| Lacrimation increased | 6 | 0.43(0.19 - 0.95) | 0.43(0.19 - 0.96) | 0.43(4.56) | 0.43(0.22-0.84) | -1.22(-2.89 - 0.45) |
| Eye pruritus | 6 | 0.39(0.18 - 0.87) | 0.39(0.18 - 0.87) | 0.39(5.66) | 0.39(0.2-0.77) | -1.35(-3.02 - 0.32) |
| Photopsia | 5 | 1.38(0.58 - 3.33) | 1.38(0.58 - 3.33) | 1.38(0.53) | 1.38(0.66-2.88) | 0.47(-1.2 - 2.13) |
| Blepharospasm | 4 | 1.23(0.46 - 3.27) | 1.23(0.46 - 3.27) | 1.23(0.17) | 1.23(0.54-2.79) | 0.29(-1.37 - 1.96) |
| Visual field defect | 4 | 1.08(0.4 - 2.87) | 1.08(0.40 - 2.87) | 1.08(0.02) | 1.08(0.47-2.45) | 0.11(-1.56 - 1.77) |
| Blindness transient | 4 | 1.30(0.49 - 3.46) | 1.30(0.49 - 3.46) | 1.30(0.27) | 1.30(0.57-2.95) | 0.38(-1.29 - 2.04) |
| Eye haemorrhage | 3 | 0.49(0.16 - 1.51) | 0.49(0.16 - 1.51) | 0.49(1.63) | 0.49(0.19-1.25) | -1.04(-2.71 - 0.63) |
| Night blindness | 3 | 4.55(1.46 - 14.15) | 4.55(1.46 - 14.15) | 4.55(8.25) | 4.53(1.75-11.70) | 2.18(0.51 - 3.85) |
| Halo vision | 3 | 5.09(1.63 - 15.84) | 5.09(1.63 - 15.84) | 5.09(9.79) | 5.06(1.96-13.09) | 2.34(0.67 - 4.01) |
| Periorbital oedema | 2 | 0.76(0.19 - 3.02) | 0.76(0.19 - 3.02) | 0.76(0.16) | 0.76(0.24-2.41) | -0.40(-2.07 - 1.26) |

Supplementary Table 2 Ocular adverse reactions male subjects treated with desvenlafaxine in PT level

| PT | a | ROR(95%Cl) | PRR(95%Cl) | PRR($\text{χ}^{\text{2}}$) | EBGM(95%Cl) | IC(95%Cl) |
| --- | --- | --- | --- | --- | --- | --- |
| Vision blurred | 34 | 2.53(1.81 - 3.55) | 2.52(1.80 - 3.53) | 2.52(31.27) | 2.52(1.90-3.34) | 1.33(-0.33 - 3.00) |
| Visual impairment | 18 | 1.75(1.10 - 2.77) | 1.74(1.10 - 2.77) | 1.74(5.71) | 1.74(1.18-2.57) | 0.80(-0.87 - 2.47) |
| Mydriasis | 10 | 7.95(4.27 - 14.81) | 7.94(4.27 - 14.79) | 7.94(60.44) | 7.91(4.7-13.31) | 2.98(1.32 - 4.65) |
| Photophobia | 8 | 4.58(2.29 - 9.17) | 4.57(2.28 - 9.16) | 4.57(22.28) | 4.56(2.55-8.16) | 2.19(0.52 - 3.86) |
| Eye pain | 6 | 1.51(0.68 - 3.35) | 1.51(0.68 - 3.35) | 1.51(1.02) | 1.51(0.77-2.94) | 0.59(-1.08 - 2.26) |
| Visual acuity reduced | 5 | 1.25(0.52 - 3.01) | 1.25(0.52 - 3.01) | 1.25(0.25) | 1.25(0.6-2.61) | 0.32(-1.34 - 1.99) |
| Blindness | 4 | 1.06(0.40 - 2.83) | 1.06(0.40 - 2.83) | 1.06(0.01) | 1.06(0.47-2.41) | 0.09(-1.58 - 1.75) |
| Photopsia | 4 | 6.31(2.36 - 16.86) | 6.31(2.36 - 16.85) | 6.31(17.81) | 6.29(2.77-14.31) | 2.65(0.98 - 4.32) |
| Abnormal sensation in eye | 3 | 8.76(2.82 - 27.23) | 8.75(2.81 - 27.22) | 8.75(20.5) | 8.71(3.37-22.52) | 3.12(1.45 - 4.79) |
| Eye disorder | 3 | 1.18(0.38 - 3.66) | 1.18(0.38 - 3.66) | 1.18(0.08) | 1.18(0.46-3.04) | 0.24(-1.43 - 1.90) |
| Diplopia | 3 | 0.94(0.30 - 2.91) | 0.94(0.30 - 2.91) | 0.94(0.01) | 0.94(0.36-2.42) | -0.09(-1.76 - 1.58) |
| Cataract | 3 | 0.63(0.2 - 1.94) | 0.63(0.20 - 1.94) | 0.63(0.67) | 0.63(0.24-1.62) | -0.67(-2.34 - 0.99) |
| Ocular hyperaemia | 2 | 0.53(0.13 - 2.10) | 0.53(0.13 - 2.11) | 0.53(0.85) | 0.53(0.17-1.68) | -0.93(-2.59 - 0.74) |
| Eye swelling | 2 | 0.72(0.18 - 2.89) | 0.72(0.18 - 2.89) | 0.72(0.21) | 0.72(0.23-2.30) | -0.47(-2.14 - 1.20) |
| Eye movement disorder | 2 | 2.73(0.68 - 10.95) | 2.73(0.68 - 10.94) | 2.73(2.20) | 2.73(0.86-8.72) | 1.45(-0.22 - 3.12) |
| Dry eye | 2 | 0.67(0.17 - 2.69) | 0.67(0.17 - 2.69) | 0.67(0.32) | 0.67(0.21-2.14) | -0.57(-2.24 - 1.09) |
| Glaucoma | 2 | 1.24(0.31 - 4.95) | 1.24(0.31 - 4.95) | 1.24(0.09) | 1.24(0.39-3.95) | 0.31(-1.36 - 1.97) |
| Periorbital oedema | 1 | 1.51(0.21 - 10.7) | 1.51(0.21 - 10.70) | 1.51(0.17) | 1.51(0.29-7.77) | 0.59(-1.08 - 2.26) |
| Metamorphopsia | 1 | 4.63(0.65 - 32.99) | 4.63(0.65 - 32.99) | 4.63(2.84) | 4.62(0.90-23.9) | 2.21(0.54 - 3.88) |
| Oculogyric crisis | 1 | 4.77(0.67 - 33.97) | 4.77(0.67 - 33.96) | 4.77(2.97) | 4.76(0.92-24.6) | 2.25(0.58 - 3.92) |
| Eye oedema | 1 | 3.82(0.54 - 27.17) | 3.82(0.54 - 27.17) | 3.82(2.08) | 3.81(0.74-19.69) | 1.93(0.26 - 3.60) |
| Eye haemorrhage | 1 | 0.76(0.11 - 5.38) | 0.76(0.11 - 5.38) | 0.76(0.08) | 0.76(0.15-3.91) | -0.40(-2.07 - 1.27) |
| Myopia | 1 | 4.75(0.67 - 33.79) | 4.75(0.67 - 33.78) | 4.75(2.95) | 4.74(0.92-24.47) | 2.24(0.57 - 3.92) |
| Asthenopia | 1 | 2.64(0.37 - 18.8) | 2.64(0.37 - 18.8) | 2.64(1.02) | 2.64(0.51-13.64) | 1.40(-0.27 - 3.07) |
| Blepharospasm | 1 | 2.71(0.38 - 19.26) | 2.71(0.38 - 19.26) | 2.71(1.08) | 2.71(0.52-13.97) | 1.44(-0.23 - 3.11) |
| Pupillary disorder | 1 | 11.63(1.63 - 83.13) | 11.63(1.63 - 83.11) | 11.63(9.65) | 11.56(2.23-59.93) | 3.53(1.85 - 5.21) |
| Macular degeneration | 1 | 1.31(0.18 - 9.28) | 1.31(0.18 - 9.28) | 1.31(0.07) | 1.31(0.25-6.73) | 0.38(-1.28 - 2.05) |

Supplementary Table 3 Ocular adverse reactions female subjects treated with duloxetine in PT level

| PT | a | ROR(95%Cl) | PRR(95%Cl) | PRR($\text{χ}^{\text{2}}$) | EBGM(95%Cl) | IC(95%Cl) |
| --- | --- | --- | --- | --- | --- | --- |
| Vision blurred | 548 | 2.16(1.98 - 2.35) | 2.15(1.98 - 2.34) | 2.15(334.98) | 2.14(1.99-2.30) | 1.10(-0.57 - 2.76) |
| Visual impairment | 247 | 1.19(1.05 - 1.35) | 1.19(1.05 - 1.35) | 1.19(7.36) | 1.19(1.07-1.32) | 0.25(-1.42 - 1.91) |
| Mydriasis | 155 | 6.81(5.80 - 8.00) | 6.80(5.80 - 7.99) | 6.80(742.19) | 6.61(5.78-7.56) | 2.73(1.06 - 4.39) |
| Eye pain | 110 | 1.15(0.95 - 1.39) | 1.15(0.95 - 1.39) | 1.15(2.19) | 1.15(0.98-1.35) | 0.20(-1.46 - 1.87) |
| Photophobia | 90 | 2.56(2.08 - 3.16) | 2.56(2.08 - 3.15) | 2.56(84.62) | 2.54(2.14-3.03) | 1.35(-0.32 - 3.01) |
| Diplopia | 75 | 1.61(1.28 - 2.02) | 1.61(1.28 - 2.02) | 1.61(17.22) | 1.61(1.33-1.94) | 0.68(-0.98 - 2.35) |
| Visual acuity reduced | 70 | 1.17(0.92 - 1.48) | 1.17(0.92 - 1.48) | 1.17(1.67) | 1.17(0.96-1.42) | 0.22(-1.44 - 1.89) |
| Dry eye | 68 | 0.81(0.64 - 1.03) | 0.81(0.64 - 1.03) | 0.81(3.08) | 0.81(0.66-0.99) | -0.31(-1.97 - 1.36) |
| Eye swelling | 58 | 0.79(0.61 - 1.03) | 0.79(0.61 - 1.03) | 0.79(3.14) | 0.79(0.64-0.98) | -0.33(-2.00 - 1.33) |
| Eye disorder | 54 | 0.97(0.74 - 1.27) | 0.97(0.74 - 1.27) | 0.97(0.05) | 0.97(0.77-1.21) | -0.05(-1.71 - 1.62) |
| Photopsia | 50 | 3.78(2.85 - 5.00) | 3.78(2.85 - 4.99) | 3.78(100.13) | 3.72(2.95-4.71) | 1.90(0.23 - 3.56) |
| Blindness | 39 | 0.69(0.51 - 0.95) | 0.69(0.51 - 0.95) | 0.69(5.24) | 0.70(0.53-0.90) | -0.52(-2.19 - 1.14) |
| Eye irritation | 39 | 0.49(0.36 - 0.67) | 0.49(0.36 - 0.67) | 0.49(20.61) | 0.49(0.38-0.64) | -1.02(-2.69 - 0.64) |
| Eye movement disorder | 38 | 3.52(2.55 - 4.85) | 3.52(2.55 - 4.85) | 3.52(67.27) | 3.47(2.66-4.54) | 1.80(0.13 - 3.46) |
| Glaucoma | 36 | 1.14(0.82 - 1.58) | 1.14(0.82 - 1.58) | 1.14(0.58) | 1.13(0.86-1.49) | 0.18(-1.48 - 1.85) |
| Retinal detachment | 34 | 2.62(1.87 - 3.68) | 2.62(1.87 - 3.68) | 2.62(33.68) | 2.60(1.96-3.45) | 1.38(-0.29 - 3.05) |
| Ocular hyperaemia | 33 | 0.45(0.32 - 0.63) | 0.45(0.32 - 0.63) | 0.45(22.44) | 0.45(0.34-0.60) | -1.16(-2.82 - 0.51) |
| Cataract | 31 | 0.30(0.21 - 0.43) | 0.30(0.21 - 0.43) | 0.30(49.73) | 0.30(0.23-0.41) | -1.72(-3.38 - -0.05) |
| Visual field defect | 27 | 1.97(1.35 - 2.87) | 1.97(1.35 - 2.87) | 1.97(12.70) | 1.96(1.42-2.69) | 0.97(-0.70 - 2.64) |
| Blepharospasm | 23 | 1.91(1.26 - 2.88) | 1.91(1.26 - 2.88) | 1.91(9.83) | 1.90(1.35-2.68) | 0.92(-0.74 - 2.59) |
| Ocular discomfort | 22 | 1.33(0.88 - 2.03) | 1.33(0.88 - 2.03) | 1.33(1.83) | 1.33(0.94-1.89) | 0.41(-1.25 - 2.08) |
| Angle closure glaucoma | 22 | 3.52(2.31 - 5.36) | 3.52(2.31 - 5.36) | 3.52(38.98) | 3.47(2.44-4.94) | 1.80(0.13 - 3.46) |
| Lacrimation increased | 22 | 0.42(0.28 - 0.64) | 0.42(0.28 - 0.64) | 0.42(17.34) | 0.42(0.30-0.60) | -1.24(-2.9 - 0.43) |
| Vitreous floaters | 21 | 1.26(0.82 - 1.94) | 1.26(0.82 - 1.94) | 1.26(1.15) | 1.26(0.88-1.81) | 0.34(-1.33 - 2.00) |
| Abnormal sensation in eye | 21 | 2.52(1.64 - 3.87) | 2.52(1.64 - 3.87) | 2.52(18.95) | 2.50(1.74-3.58) | 1.32(-0.35 - 2.99) |
| Miosis | 18 | 1.68(1.06 - 2.67) | 1.68(1.06 - 2.67) | 1.68(4.91) | 1.67(1.14-2.47) | 0.74(-0.92 - 2.41) |
| Eye pruritus | 17 | 0.30(0.19 - 0.48) | 0.30(0.19 - 0.48) | 0.30(28.05) | 0.30(0.20-0.45) | -1.74(-3.41 - -0.07) |
| Asthenopia | 15 | 1.57(0.95 - 2.61) | 1.57(0.95 - 2.61) | 1.57(3.09) | 1.57(1.02-2.40) | 0.65(-1.02 - 2.31) |
| Retinal tear | 13 | 3.29(1.90 - 5.70) | 3.29(1.90 - 5.70) | 3.29(20.41) | 3.26(2.06-5.15) | 1.70(0.03 - 3.37) |
| Blindness transient | 12 | 1.05(0.59 - 1.85) | 1.05(0.59 - 1.85) | 1.05(0.03) | 1.05(0.65-1.68) | 0.07(-1.60 - 1.73) |

Supplementary Table 4 Ocular adverse reactions male subjects treated with duloxetine in PT level

| PT | a | ROR(95%Cl) | PRR(95%Cl) | PRR($\text{χ}^{\text{2}}$) | EBGM(95%Cl) | IC(95%Cl) |
| --- | --- | --- | --- | --- | --- | --- |
| Vision blurred | 134 | 2.18(1.84 - 2.59) | 2.18(1.84 - 2.58) | 2.18(84.81) | 2.17(1.88-2.50) | 1.12(-0.55 - 2.78) |
| Visual impairment | 51 | 1.08(0.82 - 1.42) | 1.08(0.82 - 1.42) | 1.08(0.29) | 1.08(0.86-1.36) | 0.11(-1.56 - 1.77) |
| Photophobia | 30 | 3.77(2.63 - 5.40) | 3.76(2.63 - 5.39) | 3.76(60.31) | 3.74(2.77-5.05) | 1.90(0.24 - 3.57) |
| Diplopia | 22 | 1.51(0.99 - 2.29) | 1.51(0.99 - 2.29) | 1.51(3.73) | 1.50(1.06-2.14) | 0.59(-1.08 - 2.26) |
| Visual acuity reduced | 21 | 1.15(0.75 - 1.76) | 1.15(0.75 - 1.76) | 1.15(0.40) | 1.15(0.8-1.64) | 0.20(-1.47 - 1.86) |
| Eye pain | 19 | 1.04(0.66 - 1.63) | 1.04(0.66 - 1.63) | 1.04(0.03) | 1.04(0.71-1.52) | 0.06(-1.61 - 1.72) |
| Mydriasis | 19 | 3.30(2.10 - 5.19) | 3.30(2.10 - 5.19) | 3.30(30.25) | 3.28(2.25-4.79) | 1.71(0.05 - 3.38) |
| Eye disorder | 17 | 1.46(0.91 - 2.35) | 1.46(0.91 - 2.35) | 1.46(2.45) | 1.46(0.98-2.17) | 0.54(-1.12 - 2.21) |
| Glaucoma | 14 | 1.90(1.12 - 3.20) | 1.89(1.12 - 3.20) | 1.89(5.89) | 1.89(1.22-2.93) | 0.92(-0.75 - 2.59) |
| Photopsia | 13 | 4.51(2.61 - 7.79) | 4.50(2.61 - 7.78) | 4.5(35.05) | 4.46(2.83-7.06) | 2.16(0.49 - 3.83) |
| Miosis | 12 | 1.69(0.96 - 2.99) | 1.69(0.96 - 2.99) | 1.69(3.40) | 1.69(1.05-2.72) | 0.76(-0.91 - 2.42) |
| Eye movement disorder | 12 | 3.60(2.04 - 6.36) | 3.60(2.04 - 6.36) | 3.6(22.35) | 3.58(2.22-5.76) | 1.84(0.17 - 3.51) |
| Ocular hyperaemia | 11 | 0.63(0.35 - 1.14) | 0.63(0.35 - 1.14) | 0.63(2.36) | 0.63(0.39-1.04) | -0.66(-2.33 - 1.00) |
| Blindness unilateral | 9 | 1.33(0.69 - 2.56) | 1.33(0.69 - 2.56) | 1.33(0.73) | 1.33(0.77-2.30) | 0.41(-1.26 - 2.08) |
| Lacrimation increased | 9 | 0.74(0.38 - 1.41) | 0.74(0.38 - 1.41) | 0.74(0.86) | 0.74(0.43-1.27) | -0.44(-2.11 - 1.22) |
| Blindness | 8 | 0.46(0.23 - 0.93) | 0.46(0.23 - 0.93) | 0.46(4.99) | 0.46(0.26-0.83) | -1.11(-2.78 - 0.56) |
| Eye irritation | 8 | 0.55(0.27 - 1.09) | 0.55(0.27 - 1.09) | 0.55(2.99) | 0.55(0.31-0.98) | -0.87(-2.53 - 0.8) |
| Cataract | 8 | 0.36(0.18 - 0.73) | 0.36(0.18 - 0.73) | 0.36(8.87) | 0.36(0.20-0.65) | -1.45(-3.12 - 0.21) |
| Eye swelling | 7 | 0.55(0.26 - 1.16) | 0.55(0.26 - 1.16) | 0.55(2.56) | 0.55(0.30-1.03) | -0.86(-2.52 - 0.81) |
| Dry eye | 7 | 0.51(0.24 - 1.08) | 0.51(0.24 - 1.08) | 0.51(3.24) | 0.51(0.28-0.95) | -0.96(-2.63 - 0.7) |
| Eyelid oedema | 6 | 1.12(0.50 - 2.49) | 1.12(0.50 - 2.49) | 1.12(0.07) | 1.12(0.57-2.18) | 0.16(-1.51 - 1.82) |
| Blindness transient | 6 | 1.75(0.78 - 3.90) | 1.75(0.78 - 3.90) | 1.75(1.91) | 1.74(0.89-3.41) | 0.8(-0.86 - 2.47) |
| Eye pruritus | 5 | 0.47(0.20 - 1.14) | 0.47(0.20 - 1.14) | 0.47(2.92) | 0.47(0.23-0.99) | -1.08(-2.74 - 0.59) |
| Abnormal sensation in eye | 5 | 3.19(1.32 - 7.69) | 3.19(1.32 - 7.69) | 3.19(7.45) | 3.17(1.52-6.62) | 1.66(-0.01 - 3.33) |
| Altered visual depth perception | 5 | 13.95(5.72 - 34.05) | 13.95(5.72 - 34.04) | 13.95(58.01) | 13.5(6.4-28.48) | 3.75(2.07 - 5.44) |
| Retinal haemorrhage | 5 | 1.01(0.42 - 2.42) | 1.01(0.42 - 2.42) | 1.01(0.00) | 1.01(0.48-2.10) | 0.01(-1.66 - 1.68) |
| Retinal detachment | 5 | 0.87(0.36 - 2.08) | 0.87(0.36 - 2.08) | 0.87(0.10) | 0.87(0.42-1.81) | -0.21(-1.87 - 1.46) |
| Vitreous detachment | 5 | 4.48(1.85 - 10.81) | 4.48(1.85 - 10.81) | 4.48(13.35) | 4.44(2.12-9.28) | 2.15(0.48 - 3.82) |
| Exophthalmos | 4 | 4.38(1.64 - 11.74) | 4.38(1.64 - 11.74) | 4.38(10.32) | 4.34(1.9-9.91) | 2.12(0.45 - 3.79) |
| Dysmetropsia | 4 | 172.36(53.08 - 559.74) | 172.34(53.07 - 559.67) | 172.34(471.73) | 119.62(44.65-320.5) | 6.90(5.07 - 8.74) |

Supplementary Table 5 Ocular adverse reactions female subjects treated with levomilnacipran in PT level

| PT | a | ROR(95%Cl) | PRR(95%Cl) | PRR($\text{χ}^{\text{2}}$) | EBGM(95%Cl) | IC(95%Cl) |
| --- | --- | --- | --- | --- | --- | --- |
| Visual impairment | 9 | 5.99(3.11 - 11.56) | 5.93(3.07 - 11.45) | 5.93(36.97) | 5.93(3.42-10.28) | 2.57(0.90 - 4.24) |
| Vision blurred | 6 | 3.23(1.45 - 7.21) | 3.21(1.44 - 7.17) | 3.21(9.16) | 3.21(1.64-6.29) | 1.68(0.01 - 3.35) |
| Keratitis | 1 | 24.82(3.49 - 176.58) | 24.79(3.48 - 176.35) | 24.79(22.81) | 24.76(4.79-127.90) | 4.63(2.96 - 6.30) |
| Eye irritation | 1 | 1.73(0.24 - 12.29) | 1.73(0.24 - 12.29) | 1.73(0.31) | 1.73(0.33-8.92) | 0.79(-0.88 - 2.46) |
| Mydriasis | 1 | 5.85(0.82 - 41.63) | 5.85(0.82 - 41.58) | 5.85(4.02) | 5.85(1.13-30.18) | 2.55(0.88 - 4.22) |
| Cataract | 1 | 1.34(0.19 - 9.56) | 1.34(0.19 - 9.55) | 1.34(0.09) | 1.34(0.26-6.94) | 0.43(-1.24 - 2.10) |
| Ocular discomfort | 1 | 8.31(1.17 - 59.12) | 8.30(1.17 - 59.05) | 8.30(6.42) | 8.30(1.61-42.86) | 3.05(1.38 - 4.72) |
| Ocular hypertension | 1 | 51.94(7.29 - 369.91) | 51.87(7.28 - 369.42) | 51.87(49.8) | 51.78(10.02-267.62) | 5.69(4.02 - 7.37) |
| Dry eye | 1 | 1.63(0.23 - 11.60) | 1.63(0.23 - 11.60) | 1.63(0.24) | 1.63(0.32-8.42) | 0.71(-0.96 - 2.38) |
| Angle closure glaucoma | 1 | 21.70(3.05 - 154.35) | 21.67(3.05 - 154.15) | 21.67(19.70) | 21.65(4.19-111.81) | 4.44(2.77 - 6.11) |
| Eye haemorrhage | 1 | 5.99(0.84 - 42.60) | 5.99(0.84 - 42.56) | 5.99(4.15) | 5.98(1.16-30.89) | 2.58(0.91 - 4.25) |

Supplementary Table 6 Ocular adverse reactions male subjects treated with levomilnacipran in PT level

| PT | a | ROR(95%Cl) | PRR(95%Cl) | PRR($\text{χ}^{\text{2}}$) | EBGM(95%Cl) | IC(95%Cl) |
| --- | --- | --- | --- | --- | --- | --- |
| Photophobia | 1 | 13.74(1.93 - 97.95) | 13.70(1.92 - 97.62) | 13.70(11.77) | 13.69(2.65-70.82) | 3.78(2.10 - 5.45) |
| Eye swelling | 1 | 8.69(1.22 - 61.93) | 8.66(1.22 - 61.74) | 8.66(6.78) | 8.66(1.68-44.79) | 3.11(1.44 - 4.79) |
| Retinal detachment | 1 | 19.13(2.68 - 136.33) | 19.06(2.67 - 135.87) | 19.06(17.11) | 19.05(3.68-98.56) | 4.25(2.58 - 5.93) |
| Visual impairment | 1 | 2.33(0.33 - 16.59) | 2.32(0.33 - 16.56) | 2.32(0.76) | 2.32(0.45-12.02) | 1.22(-0.46 - 2.89) |
| Eye pain | 1 | 6.04(0.85 - 43.04) | 6.02(0.85 - 42.91) | 6.02(4.19) | 6.02(1.16-31.14) | 2.59(0.91 - 4.27) |

Supplementary Table 7 Ocular adverse reactions female subjects treated with milnacipran in PT level

| PT | a | ROR(95%Cl) | PRR(95%Cl) | PRR($\text{χ}^{\text{2}}$) | EBGM(95%Cl) | IC(95%Cl) |
| --- | --- | --- | --- | --- | --- | --- |
| Vision blurred | 40 | 3.32(2.43 - 4.54) | 3.30(2.42 - 4.51) | 3.30(64.41) | 3.30(2.55-4.29) | 1.72(0.06 - 3.39) |
| Visual impairment | 10 | 1.02(0.55 - 1.89) | 1.02(0.55 - 1.89) | 1.02(0.00) | 1.02(0.61-1.71) | 0.02(-1.64 - 1.69) |
| Eye swelling | 4 | 1.16(0.43 - 3.09) | 1.16(0.43 - 3.09) | 1.16(0.09) | 1.16(0.51-2.63) | 0.21(-1.46 - 1.88) |
| Eye pain | 3 | 0.66(0.21 - 2.06) | 0.66(0.21 - 2.06) | 0.66(0.51) | 0.66(0.26-1.71) | -0.59(-2.26 - 1.08) |
| Ocular hyperaemia | 3 | 0.86(0.28 - 2.68) | 0.86(0.28 - 2.68) | 0.86(0.06) | 0.86(0.33-2.23) | -0.21(-1.88 - 1.45) |
| Mydriasis | 3 | 2.71(0.87 - 8.41) | 2.71(0.87 - 8.40) | 2.71(3.23) | 2.71(1.05-6.98) | 1.44(-0.23 - 3.10) |
| Dry eye | 2 | 0.50(0.13 - 2.01) | 0.50(0.13 - 2.01) | 0.50(0.98) | 0.50(0.16-1.61) | -0.99(-2.66 - 0.68) |
| Visual acuity reduced | 2 | 0.70(0.18 - 2.82) | 0.70(0.18 - 2.82) | 0.70(0.25) | 0.70(0.22-2.25) | -0.50(-2.17 - 1.16) |
| Photophobia | 2 | 1.19(0.30 - 4.78) | 1.19(0.30 - 4.78) | 1.19(0.06) | 1.19(0.37-3.81) | 0.26(-1.41 - 1.92) |
| Eye pruritus | 2 | 0.74(0.19 - 2.98) | 0.74(0.19 - 2.98) | 0.74(0.18) | 0.74(0.23-2.38) | -0.43(-2.09 - 1.24) |
| Eye haemorrhage | 2 | 1.85(0.46 - 7.39) | 1.85(0.46 - 7.39) | 1.85(0.78) | 1.85(0.58-5.89) | 0.89(-0.78 - 2.55) |
| Lacrimation increased | 1 | 0.41(0.06 - 2.89) | 0.41(0.06 - 2.89) | 0.41(0.86) | 0.41(0.08-2.10) | -1.3(-2.96 - 0.37) |
| Blindness unilateral | 1 | 1.03(0.14 - 7.29) | 1.03(0.14 - 7.29) | 1.03(0.00) | 1.03(0.2-5.29) | 0.04(-1.63 - 1.70) |
| Visual field defect | 1 | 1.53(0.22 - 10.89) | 1.53(0.22 - 10.89) | 1.53(0.19) | 1.53(0.3-7.91) | 0.62(-1.05 - 2.28) |
| Glaucoma | 1 | 0.67(0.09 - 4.73) | 0.67(0.09 - 4.73) | 0.67(0.17) | 0.67(0.13-3.44) | -0.58(-2.25 - 1.08) |
| Myopia | 1 | 5.36(0.75 - 38.08) | 5.36(0.75 - 38.08) | 5.36(3.54) | 5.35(1.04-27.62) | 2.42(0.75 - 4.09) |
| Photopsia | 1 | 1.58(0.22 - 11.19) | 1.58(0.22 - 11.19) | 1.58(0.21) | 1.58(0.31-8.13) | 0.66(-1.01 - 2.32) |
| Eyelid margin crusting | 1 | 4.93(0.69 - 35.06) | 4.93(0.69 - 35.05) | 4.93(3.13) | 4.93(0.95-25.42) | 2.30(0.63 - 3.97) |
| Abnormal sensation in eye | 1 | 2.52(0.35 - 17.88) | 2.52(0.35 - 17.88) | 2.52(0.91) | 2.52(0.49-12.98) | 1.33(-0.34 - 3.00) |
| Vitreous floaters | 1 | 1.27(0.18 - 9.04) | 1.27(0.18 - 9.04) | 1.27(0.06) | 1.27(0.25-6.56) | 0.35(-1.32 - 2.01) |
| Blepharospasm | 1 | 1.75(0.25 - 12.41) | 1.75(0.25 - 12.4) | 1.75(0.32) | 1.75(0.34-9.00) | 0.80(-0.86 - 2.47) |
| Diplopia | 1 | 0.45(0.06 - 3.21) | 0.45(0.06 - 3.22) | 0.45(0.66) | 0.45(0.09-2.34) | -1.14(-2.81 - 0.52) |
| Eye movement disorder | 1 | 1.93(0.27 - 13.74) | 1.93(0.27 - 13.74) | 1.93(0.45) | 1.93(0.37-9.97) | 0.95(-0.72 - 2.62) |
| Eye irritation | 1 | 0.27(0.04 - 1.89) | 0.27(0.04 - 1.89) | 0.27(2.02) | 0.27(0.05-1.38) | -1.91(-3.57 - -0.24) |
| Altered visual depth perception | 1 | 13.21(1.85 - 94.08) | 13.21(1.85 - 94.06) | 13.21(11.25) | 13.17(2.55-68.07) | 3.72(2.04 - 5.39) |
| Eye discharge | 1 | 1.21(0.17 - 8.61) | 1.21(0.17 - 8.61) | 1.21(0.04) | 1.21(0.23-6.25) | 0.28(-1.39 - 1.94) |
| Eye disorder | 1 | 0.38(0.05 - 2.69) | 0.38(0.05 - 2.70) | 0.38(1.01) | 0.38(0.07-1.96) | -1.40(-3.06 - 0.27) |
| Accommodation disorder | 1 | 15.54(2.18 - 110.74) | 15.54(2.18 - 110.72) | 15.54(13.55) | 15.48(2.99-80.07) | 3.95(2.28 - 5.63) |

Supplementary Table 8 Ocular adverse reactions male subjects treated with milnacipran in PT level

| PT | a | ROR(95%Cl) | PRR(95%Cl) | PRR($\text{χ}^{\text{2}}$) | EBGM(95%Cl) | IC(95%Cl) |
| --- | --- | --- | --- | --- | --- | --- |
| Visual impairment | 2 | 1.74(0.44 - 6.99) | 1.74(0.44 - 6.98) | 1.74(0.63) | 1.74(0.55-5.57) | 0.80(-0.87 - 2.47) |
| Visual acuity reduced transiently | 1 | 211.15(29.32 - 1520.44) | 210.87(29.29 - 1518.40) | 210.87(206.17) | 208.15(39.9-1085.82) | 7.70(6.00 - 9.40) |
| Amaurosis | 1 | 56.70(7.95 - 404.49) | 56.63(7.94 - 403.96) | 56.63(54.46) | 56.43(10.9-292.09) | 5.82(4.14 - 7.5) |
| Oscillopsia | 1 | 291.78(40.32 - 2111.25) | 291.39(40.27 - 2108.42) | 291.39(284.22) | 286.20(54.64-1499.12) | 8.16(6.45 - 9.87) |
| Blepharospasm | 1 | 24.42(3.43 - 173.89) | 24.39(3.43 - 173.66) | 24.39(22.40) | 24.36(4.71-125.86) | 4.61(2.93 - 6.28) |
| Symblepharon | 1 | 232.58(32.26 - 1676.86) | 232.27(32.21 - 1674.61) | 232.27(226.98) | 228.96(43.84-1195.7) | 7.84(6.13 - 9.54) |
| Punctate keratitis | 1 | 88.66(12.4 - 633.68) | 88.54(12.39 - 632.83) | 88.54(86.08) | 88.06(16.99-456.54) | 6.46(4.78 - 8.14) |
| Eyelid oedema | 1 | 7.67(1.08 - 54.57) | 7.66(1.08 - 54.51) | 7.66(5.79) | 7.66(1.48-39.55) | 2.94(1.27 - 4.61) |
| Eye movement disorder | 1 | 12.31(1.73 - 87.61) | 12.30(1.73 - 87.50) | 12.30(10.37) | 12.29(2.38-63.47) | 3.62(1.95 - 5.29) |

Supplementary Table 9 Ocular adverse reactions female subjects treated with venlafaxine in PT level

| PT | a | ROR(95%Cl) | PRR(95%Cl) | PRR($\text{χ}^{\text{2}}$) | EBGM(95%Cl) | IC(95%Cl) |
| --- | --- | --- | --- | --- | --- | --- |
| Vision blurred | 334 | 1.65(1.48 - 1.84 ) | 1.65(1.48 - 1.84 ) | 1.65(85.16 ) | 1.65(1.5-1.8) | 0.72(-0.95 - 2.38 ) |
| Mydriasis | 216 | 12.17(10.62 - 13.96 ) | 12.14(10.59 - 13.92 ) | 12.14(2107.46 ) | 11.63(10.37-13.04) | 3.54(1.87 - 5.21 ) |
| Visual impairment | 171 | 1.04(0.89 - 1.21 ) | 1.04(0.89 - 1.21 ) | 1.04(0.23 ) | 1.04(0.91-1.18) | 0.05(-1.61 - 1.72 ) |
| Photophobia | 96 | 3.46(2.83 - 4.23 ) | 3.46(2.82 - 4.23 ) | 3.46(165.28 ) | 3.42(2.89-4.05) | 1.77(0.11 - 3.44 ) |
| Eye pain | 86 | 1.14(0.92 - 1.40 ) | 1.14(0.92 - 1.40 ) | 1.14(1.40 ) | 1.14(0.95-1.36) | 0.18(-1.48 - 1.85 ) |
| Diplopia | 54 | 1.46(1.12 - 1.91 ) | 1.46(1.12 - 1.91 ) | 1.46(7.83 ) | 1.46(1.17-1.83) | 0.55(-1.12 - 2.21 ) |
| Blindness | 38 | 0.85(0.62 - 1.17 ) | 0.85(0.62 - 1.18 ) | 0.85(0.94 ) | 0.85(0.65-1.12) | -0.23(-1.89 - 1.44 ) |
| Dry eye | 33 | 0.49(0.35 - 0.70 ) | 0.49(0.35 - 0.70 ) | 0.49(17.02 ) | 0.5(0.37-0.66) | -1.01(-2.68 - 0.65 ) |
| Photopsia | 33 | 3.13(2.22 - 4.41 ) | 3.13(2.22 - 4.41 ) | 3.13(47.20 ) | 3.10(2.33-4.13) | 1.63(-0.03 - 3.30 ) |
| Eye disorder | 32 | 0.72(0.51 - 1.02 ) | 0.72(0.51 - 1.02 ) | 0.72(3.36 ) | 0.72(0.54-0.97) | -0.46(-2.13 - 1.20 ) |
| Eye swelling | 31 | 0.53(0.38 - 0.76 ) | 0.53(0.38 - 0.76 ) | 0.53(12.58 ) | 0.54(0.4-0.72) | -0.9(-2.57 - 0.76 ) |
| Eye movement disorder | 28 | 3.26(2.25 - 4.73 ) | 3.26(2.24 - 4.73 ) | 3.26(43.29 ) | 3.23(2.36-4.41) | 1.69(0.02 - 3.36 ) |
| Cataract | 27 | 0.33(0.23 - 0.49 ) | 0.33(0.23 - 0.49 ) | 0.33(36.02 ) | 0.33(0.24-0.46) | -1.58(-3.25 - 0.08 ) |
| Glaucoma | 25 | 0.99(0.67 - 1.47 ) | 0.99(0.67 - 1.47 ) | 0.99(0.00 ) | 0.99(0.72-1.38) | -0.01(-1.67 - 1.66 ) |
| Visual acuity reduced | 23 | 0.48(0.32 - 0.73 ) | 0.48(0.32 - 0.73 ) | 0.48(12.73 ) | 0.48(0.34-0.68) | -1.05(-2.71 - 0.62 ) |
| Miosis | 19 | 2.24(1.43 - 3.52 ) | 2.24(1.43 - 3.52 ) | 2.24(12.94 ) | 2.23(1.53-3.25) | 1.16(-0.51 - 2.82 ) |
| Asthenopia | 18 | 2.39(1.50 - 3.80 ) | 2.39(1.50 - 3.80 ) | 2.39(14.36 ) | 2.37(1.61-3.5) | 1.25(-0.42 - 2.91 ) |
| Accommodation disorder | 17 | 16.68(10.21 - 27.26 ) | 16.68(10.21 - 27.25 ) | 16.68(234.99 ) | 15.7(10.41-23.68) | 3.97(2.30 - 5.65 ) |
| Eyelid oedema | 16 | 0.76(0.47 - 1.24 ) | 0.76(0.47 - 1.24 ) | 0.76(1.19 ) | 0.76(0.51-1.15) | -0.39(-2.06 - 1.28 ) |
| Ocular hyperaemia | 16 | 0.27(0.17 - 0.45 ) | 0.27(0.17 - 0.45 ) | 0.27(30.77 ) | 0.27(0.18-0.41) | -1.86(-3.53 - -0.20 ) |
| Ocular hypertension | 16 | 7.84(4.76 - 12.89 ) | 7.83(4.76 - 12.89 ) | 7.83(92.51 ) | 7.63(5.03-11.57) | 2.93(1.26 - 4.60 ) |
| Blepharospasm | 15 | 1.57(0.94 - 2.60 ) | 1.57(0.94 - 2.60 ) | 1.57(3.05 ) | 1.56(1.02-2.39) | 0.64(-1.02 - 2.31 ) |
| Abnormal sensation in eye | 14 | 2.11(1.25 - 3.57 ) | 2.11(1.25 - 3.57 ) | 2.11(8.12 ) | 2.10(1.35-3.26) | 1.07(-0.6 - 2.74 ) |
| Angle closure glaucoma | 12 | 2.41(1.36 - 4.25 ) | 2.41(1.36 - 4.25 ) | 2.41(9.76 ) | 2.39(1.49-3.85) | 1.26(-0.41 - 2.93 ) |
| Visual field defect | 12 | 1.10(0.62 - 1.94 ) | 1.10(0.62 - 1.94 ) | 1.10(0.10 ) | 1.1(0.68-1.76) | 0.13(-1.53 - 1.80 ) |
| Lacrimation increased | 11 | 0.27(0.15 - 0.48 ) | 0.27(0.15 - 0.48 ) | 0.27(22.18 ) | 0.27(0.16-0.44) | -1.90(-3.57 - -0.24 ) |
| Pupil fixed | 11 | 4.80(2.64 - 8.71 ) | 4.80(2.64 - 8.71 ) | 4.80(32.44 ) | 4.73(2.87-7.79) | 2.24(0.57 - 3.91 ) |
| Ocular discomfort | 11 | 0.84(0.46 - 1.52 ) | 0.84(0.46 - 1.52 ) | 0.84(0.33 ) | 0.84(0.51-1.38) | -0.25(-1.92 - 1.42 ) |
| Anisocoria | 11 | 32.51(17.35 - 60.93 ) | 32.51(17.35 - 60.92 ) | 32.51(297.43 ) | 28.9(17.09-48.88) | 4.85(3.16 - 6.54 ) |
| Retinal tear | 9 | 2.87(1.49 - 5.53 ) | 2.87(1.49 - 5.53 ) | 2.87(10.81 ) | 2.84(1.64-4.93) | 1.51(-0.16 - 3.18 ) |

Supplementary Table 10 Ocular adverse reactions male subjects treated with venlafaxine in PT level

| PT | a | ROR(95%Cl) | PRR(95%Cl) | PRR($\text{χ}^{\text{2}}$) | EBGM(95%Cl) | IC(95%Cl) |
| --- | --- | --- | --- | --- | --- | --- |
| Vision blurred | 90 | 1.45(1.18 - 1.79) | 1.45(1.18 - 1.78) | 1.45(12.56) | 1.45(1.22-1.72) | 0.53(-1.13 - 2.20) |
| Mydriasis | 63 | 11.13(8.66 - 14.30) | 11.11(8.64 - 14.27) | 11.11(563.28) | 10.82(8.78-13.35) | 3.44(1.77 - 5.10) |
| Visual impairment | 46 | 0.97(0.72 - 1.29) | 0.97(0.72 - 1.29) | 0.97(0.05) | 0.97(0.76-1.23) | -0.05(-1.71 - 1.62) |
| Miosis | 40 | 5.68(4.15 - 7.76) | 5.67(4.15 - 7.75) | 5.67(151.74) | 5.6(4.32-7.28) | 2.49(0.82 - 4.15) |
| Photophobia | 20 | 2.49(1.60 - 3.86) | 2.49(1.60 - 3.86) | 2.49(17.67) | 2.48(1.71-3.58) | 1.31(-0.36 - 2.98) |
| Eye pain | 18 | 0.98(0.62 - 1.56) | 0.98(0.62 - 1.56) | 0.98(0.01) | 0.98(0.67-1.44) | -0.03(-1.70 - 1.64) |
| Diplopia | 17 | 1.16(0.72 - 1.86) | 1.16(0.72 - 1.86) | 1.16(0.36) | 1.16(0.78-1.72) | 0.21(-1.46 - 1.88) |
| Eye movement disorder | 13 | 3.88(2.25 - 6.71) | 3.88(2.25 - 6.71) | 3.88(27.55) | 3.85(2.44-6.09) | 1.95(0.28 - 3.61) |
| Eye disorder | 11 | 0.94(0.52 - 1.69) | 0.94(0.52 - 1.69) | 0.94(0.05) | 0.94(0.57-1.54) | -0.09(-1.76 - 1.57) |
| Retinopathy | 10 | 4.68(2.51 - 8.73) | 4.68(2.51 - 8.73) | 4.68(28.58) | 4.64(2.75-7.81) | 2.21(0.54 - 3.88) |
| Retinal tear | 10 | 8.00(4.28 - 14.97) | 8.00(4.28 - 14.96) | 8.00(60.00) | 7.86(4.65-13.27) | 2.97(1.3 - 4.64) |
| Visual acuity reduced | 9 | 0.49(0.25 - 0.94) | 0.49(0.25 - 0.94) | 0.49(4.82) | 0.49(0.28-0.84) | -1.03(-2.7 - 0.63) |
| Vitreous opacities | 9 | 13.25(6.82 - 25.75) | 13.24(6.81 - 25.74) | 13.24(98.5) | 12.84(7.36-22.39) | 3.68(2.01 - 5.36) |
| Retinopathy hypertensive | 8 | 39.05(18.87 - 80.82) | 39.04(18.87 - 80.8) | 39.04(269.29) | 35.55(19.34-65.33) | 5.15(3.46 - 6.85) |
| Vitreous degeneration | 8 | 123.41(55.66 - 273.63) | 123.38(55.65 - 273.56) | 123.38(735.69) | 93.71(48.13-182.45) | 6.55(4.81 - 8.29) |
| Blindness | 7 | 0.40(0.19 - 0.84) | 0.40(0.19 - 0.84) | 0.40(6.2) | 0.40(0.22-0.75) | -1.31(-2.98 - 0.36) |
| Accommodation disorder | 7 | 17.76(8.32 - 37.89) | 17.76(8.32 - 37.88) | 17.76(105.82) | 17.02(9.03-32.08) | 4.09(2.41 - 5.77) |
| Angle closure glaucoma | 7 | 6.23(2.95 - 13.16) | 6.23(2.95 - 13.15) | 6.23(30.27) | 6.15(3.29-11.49) | 2.62(0.95 - 4.29) |
| Glaucoma | 6 | 0.81(0.36 - 1.79) | 0.81(0.36 - 1.79) | 0.81(0.28) | 0.81(0.41-1.57) | -0.31(-1.98 - 1.35) |
| Blindness unilateral | 5 | 0.73(0.3 - 1.76) | 0.73(0.30 - 1.76) | 0.73(0.49) | 0.73(0.35-1.53) | -0.45(-2.11 - 1.22) |
| Asthenopia | 5 | 2.88(1.2 - 6.95) | 2.88(1.20 - 6.95) | 2.88(6.10) | 2.87(1.37-5.99) | 1.52(-0.15 - 3.19) |
| Eyelid oedema | 5 | 0.92(0.38 - 2.22) | 0.92(0.38 - 2.22) | 0.92(0.03) | 0.92(0.44-1.93) | -0.11(-1.78 - 1.55) |
| Night blindness | 4 | 5.07(1.89 - 13.61) | 5.07(1.89 - 13.61) | 5.07(12.91) | 5.02(2.20-11.46) | 2.33(0.65 - 4) |
| Eye irritation | 4 | 0.27(0.1 - 0.72) | 0.27(0.1 - 0.72) | 0.27(7.80) | 0.27(0.12-0.62) | -1.88(-3.54 - -0.21) |
| Visual field defect | 4 | 0.9(0.34 - 2.41) | 0.90(0.34 - 2.41) | 0.90(0.04) | 0.90(0.4-2.06) | -0.14(-1.81 - 1.52) |
| Cataract | 4 | 0.18(0.07 - 0.48) | 0.18(0.07 - 0.48) | 0.18(14.82) | 0.18(0.08-0.41) | -2.46(-4.13 - -0.8) |
| Eye pruritus | 4 | 0.38(0.14 - 1.00) | 0.38(0.14 - 1.00) | 0.38(4.12) | 0.38(0.17-0.86) | -1.41(-3.07 - 0.26) |
| Blepharospasm | 4 | 2.36(0.88 - 6.30) | 2.36(0.88 - 6.3) | 2.36(3.11) | 2.35(1.03-5.35) | 1.23(-0.44 - 2.9) |
| Macular oedema | 4 | 1.11(0.42 - 2.96) | 1.11(0.42 - 2.96) | 1.11(0.04) | 1.11(0.49-2.53) | 0.15(-1.52 - 1.82) |
| Lacrimation increased | 3 | 0.24(0.08 - 0.75) | 0.24(0.08 - 0.76) | 0.24(7.05) | 0.24(0.09-0.63) | -2.04(-3.7 - -0.37) |
