# Supplementary figures and images for: Analysis of ocular adverse events associated with SNRIs
Source: Front Pharmacol. 2026 May 29;17:1837592. doi: 10.3389/fphar.2026.1837592 (PMC13260375; doi:10.3389/fphar.2026.1837592)

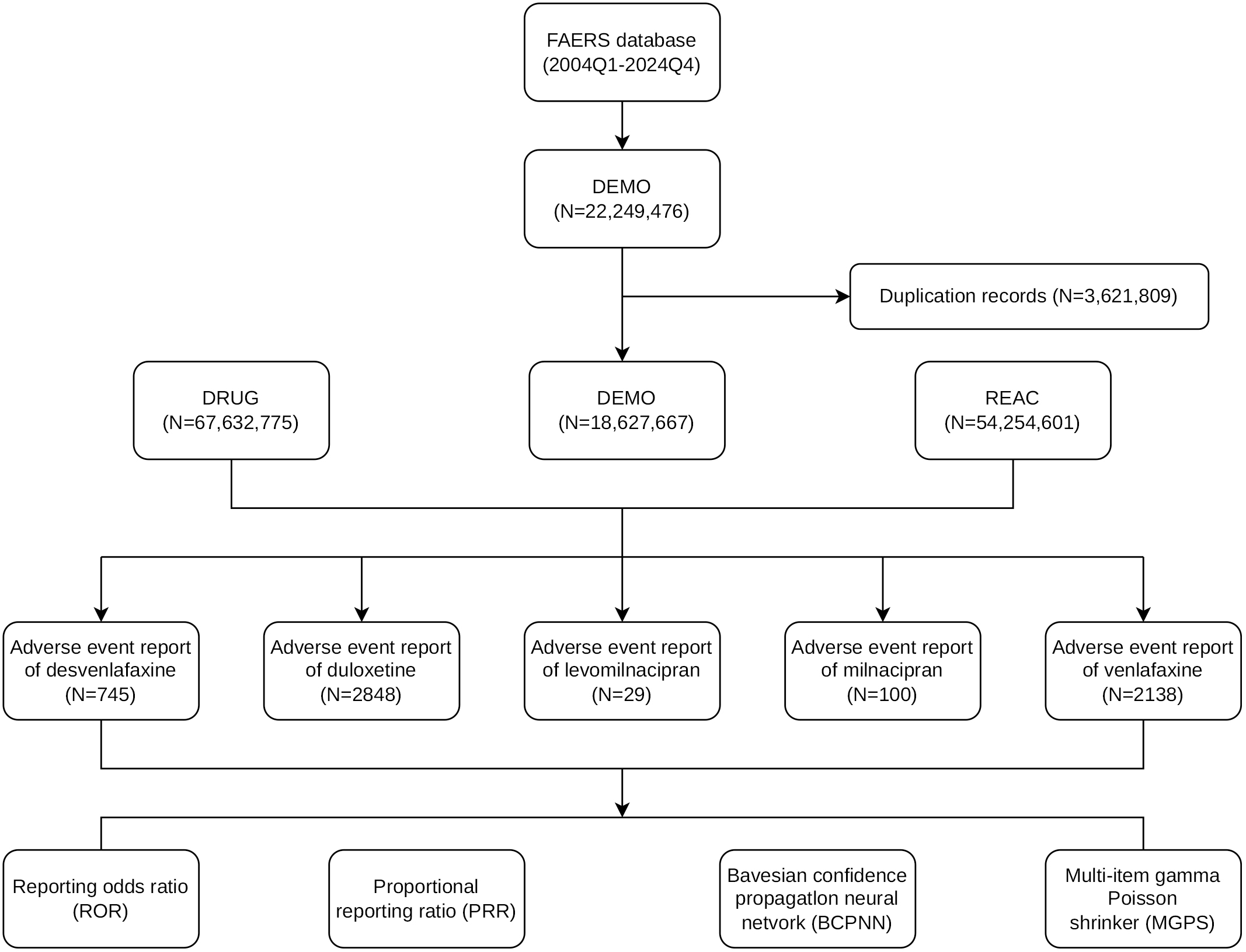

Supplement: Supplementary file 2 [file Image1.jpeg]
